# Watkins wheat landraces decode nitrogen-driven biomass trade-offs: GWAS exposes root-shoot dialectics and elite landraces for resilient agriculture

**DOI:** 10.3389/fpls.2025.1603577

**Published:** 2025-05-23

**Authors:** Abdul Waheed, Muhammad Shahid Iqbal, Zareen Sarfraz, Junliang Hou, Yanping Wei, Bo Song, Shifeng Cheng

**Affiliations:** Guangdong Laboratory for Lingnan Modern Agriculture, Genome Analysis Laboratory of the Ministry of Agriculture, Agricultural Genomics Institute at Shenzhen, Chinese Academy of Agricultural Sciences, Shenzhen, China

**Keywords:** abiotic stress, nitrogen use efficiency, root-shoot trade-offs, molecular mechanisms, crop productivity, geographic adaptation

## Abstract

**Introduction:**

Nitrogen limitation is a critical abiotic stressor that disrupts the balance between plants and their environment, imposing trade-offs in biomass allocation that threaten crop productivity and food security. While modern breeding programs often focus on improving shoot performance, the genetic mechanisms that coordinate root-shoot responses under nitrogen stress remain poorly understood. This study aimed to dissect the molecular and physiological foundations of nitrogen-driven resilience in wheat, leveraging the genetically diverse Watkins wheat landraces as a source of adaptive alleles.

**Methods:**

A total of 308 Watkins wheat landraces were phenotyped under low nitrogen (LN) and normal nitrogen (NN) conditions to assess root-shoot allocation strategies. Genome-wide association studies (GWAS) were conducted to identify candidate genes governing nitrogen-responsive traits. Functional annotation and transcriptomic validation were used to elucidate gene networks, and haplotype mapping was employed to link allelic variation to geographic adaptation. Multivariate analysis was performed to classify biomass allocation strategies among the landraces.

**Results:**

Phenotypic analysis revealed stark differences in root-shoot allocation strategies under LN and NN conditions. GWAS identified 130 candidate genes, including root-specific *RALF33* and shoot-prioritizing *TaNAR1*, involved in nitrogen-responsive traits. Functional studies highlighted antagonistic gene networks, such as *TAF6* and *TaAPY6*, balancing root meristem activity and stress adaptation. Adaptive alleles of *RALF33* in European landraces optimized root proliferation under LN, while Eurasian landraces exhibited shoot-root coordination under NN through *TaNAR1* variants. Multivariate analysis classified landraces into four distinct biomass allocation strategies, identifying elite genotypes resilient to nitrogen limitation.

**Discussion:**

By integrating genomics, phenomics, and haplotype mapping, this study connects molecular mechanisms underlying nutrient stress with ecophysiological adaptation. Key genes, such as *RALF33* and *TaAPY6*, emerged as actionable targets for marker-assisted breeding to develop nitrogen-efficient wheat varieties. These findings highlight the potential of evolutionary-informed genetics in the Watkins landraces to enhance stress resilience, providing a roadmap for sustainable crop design in the context of global nutrient scarcity.

## Introduction

The genetic bottlenecks resulting from the domestication of common wheat around 8,000 years ago and modern breeding practices that rely on a narrow set of parent lines have restricted genetic diversity (Cox, 1997; Lopes et al., 2015; Kabbaj et al., 2017). This lack of diversity limits wheat’s ability to adapt to changing environments and optimize resource allocation between root-shoot biomass (Chapman et al., 2012; Lopes et al., 2015). Addressing these bottlenecks through the use of diverse genetic resources, such as landraces, is essential for improving traits like root architecture, shoot growth, and nitrogen utilization (Rasheed et al., 2018; Marone et al., 2021). The Watkins Collection, a set of hexaploid wheat landraces gathered in the 1930s from 32 countries across Asia, Europe, and Africa, represents a unique genetic resource predating modern intensive breeding practices (Wingen et al., 2014). However, despite their geographic and genetic diversity, no study has systematically dissected how these landraces modulate root-shoot biomass allocation under nitrogen limitation. These landraces bridge the genetic divide between wild wheat progenitors and modern cultivars, harboring allelic diversity lost during post-Green Revolution breeding but critical for nitrogen adaptation (Wingen et al., 2014; Cheng et al., 2024). This geographic diversity likely encodes distinct nitrogen adaptation strategies, as landraces evolved under varied agroecological pressures such as European accessions in nitrogen-poor soils versus Eurasian lines in moderate-fertility regions (Wingen et al., 2014). Nitrogen, a cornerstone of wheat growth and productivity, epitomizes this challenge: while indispensable for chlorophyll synthesis and biomass accumulation, its inefficient utilization has precipitated an environmental crisis (Duan et al., 2019). Nitrogen availability directly modulates root-shoot resource partitioning, yet the genetic mechanisms governing this trade-off under contrasting nitrogen regimes remain poorly resolved (Mălinaş et al., 2022). By integrating Genome wide association studies (GWAS) with phenotypic analyses, we aim to identify genetic loci that regulate nitrogen utilization and biomass allocation, providing a foundation for developing wheat varieties with enhanced resource-use efficiency and resilience to environmental stressors (Hawkesford, 2014). The use of GWAS has become an indispensable tool for identifying genetic loci associated with agronomically important traits in wheat, including those related to root and shoot development and nitrogen response (Li et al., 2019; Koua et al., 2024). GWAS can help uncover the complex genetic architecture of traits such as nitrogen utilization, root development, and shoot growth by linking these traits to specific SNPs (Shi et al., 2022). Although dissecting individual traits through GWAS can provide valuable information, the integration of multiple traits such as root and shoot development and nitrogen productivity is essential for improving wheat breeding (Shi et al., 2022; Zhao et al., 2023). Notably, existing GWAS efforts in wheat have focused almost exclusively on modern cultivars or synthetic hybrids, neglecting landrace collections like Watkins. The heritable covariation between different traits suggests that genetic networks governing multiple traits can be exploited to select independent genes with pyramiding potential or pleiotropic genes with major effects (Li et al., 2019; Dwivedi et al., 2024). Root traits, such as root length, root biomass, are key determinants of a plant’s ability to acquire nutrients, particularly under nutrient-limited conditions (Wang et al., 2016; Kramer-Walter and Laughlin, 2017). Early seedling-stage dynamics are critical for establishing these allocation patterns, yet most studies focus on mature plants, overlooking developmental plasticity during this foundational phase (Postma et al., 2014). This oversight is especially pronounced in landrace germplasm like the Watkins Collection. This gap limits our ability to harness their evolutionary solutions for improving nitrogen resilience. Shoots are important for photosynthesis and nutrient transport, but early root growth plays a crucial role in establishing a strong foundation for nutrient uptake (Gu et al., 2018). Optimizing root-shoot biomass allocation under nitrogen limitation is central to improving ecological resilience in these environments (Anas et al., 2020). While several definitions of nitrogen utilization and productivity have been proposed in the literature (Xu et al., 2012; Han et al., 2015; Lammerts Van Bueren and Struik, 2017). These trade-offs are often governed by antagonistic gene networks that differentially regulate root and shoot growth under nutrient stress, a dynamic yet underexplored aspect of nitrogen adaptation. By leveraging this genetic diversity, we can uncover novel loci associated with the competitive dynamics between root-shoot biomass, providing insights into how wheat balances resource allocation during early growth stages. Understanding the genetic competition in root-shoot biomass, governed by antagonistic gene networks that balance root and shoot growth under nitrogen stress, is essential for improving wheat’s nitrogen utilization and productivity. These trade-offs are mediated by antagonistic gene networks, such as *SnRK1β3* balancing energy allocation and *OHP1* optimizing photosynthetic efficiency, which remain uncharacterized in landrace germplasm (Islam et al., 2021; Sharma et al., 2021). This study addresses a critical gap in the existing literature by focusing on the underexplored area of root-shoot biomass trade-offs in Watkins landraces under nitrogen stress. While extensive research has been conducted on various crop species and their responses to nutrient limitations, there is a notable absence of studies specifically examining how Watkins landraces a valuable genetic resource for wheat improvement allocate biomass between roots and shoots when subjected to nitrogen-deficient conditions (Sprunger et al., 2018; Brasier et al., 2019; Luo and Zhou, 2019; Su et al., 2019). Here, we investigate root-shoot biomass allocation in Watkins wheat landraces under nitrogen stress, focusing on the seedling stage a critical period for establishing nutrient uptake and growth trajectories. By integrating GWAS with phenotypic analyses, we identify genetic loci governing nitrogen utilization and biomass allocation. Our findings reveal geographic adaptation patterns, with European landraces prioritizing root investment under nitrogen limitation and Eurasian accessions optimizing shoot-root coordination under sufficient nitrogen, reflecting divergent agroecological selection pressures. These insights into the genetic architecture of nitrogen-driven trade-offs not only address a critical gap in understanding plant-environment interactions but also highlight the untapped potential of Watkins landraces for developing resilient, nitrogen-efficient wheat varieties. Ultimately, this work advances strategies to enhance food security and sustainable agriculture in nitrogen-limited and climate-variable environments.

## Materials and methods

### Plant materials

A comprehensive panel of 308 Watkins wheat landraces, meticulously collected from the Shifeng Cheng lab at the Agricultural Genomic Institute at Shenzhen, Chinese Academy of Agricultural Sciences https://wwwg2b.com/seedStor. A detailed information on these landraces is available in **Supplementary Table S1**. These accessions are distinct from modern wheat landraces, offering a rich genetic repository invaluable for wheat breeding initiatives. Unlike contemporary wheat varieties, which often exhibit reduced genetic variability due to selective breeding practices aimed at enhancing specific traits, the Watkins panel provides a broader genetic base. However, limited data is currently available on these accessions. This lack of comprehensive information presents both a challenge and an opportunity for researchers.

### Growth conditions

All experiments were conducted under controlled hydroponic conditions in growth chambers at the Agricultural Genomics Institute at Shenzhen. The experiment was repeated four times across seasonal cycles to account for environmental variability. Seeds were surface-sterilized with 2% H_2_O_2_ for 30 minutes, rinsed with distilled water, and germinated using a paper roll method. After 5 days, seedlings were transferred to 96-well blue plastic boxes. Growth parameters were rigorously maintained: temperature 25/22°C (day/night), light intensity 300 µmol m^2^ s^1^ (10/14-hour dark/light cycle), relative humidity 65–70%, and nutrient solution pH 6.0–6.5. Nitrogen treatments included low nitrogen (LN: 0.2 mM) and normal nitrogen (NN: 1 mM), applied via modified Hoagland solutions (**Table 1**). Calcium concentrations were adjusted to 2.4 mM (LN) and 1.5 mM (NN) using CaCl_2_·4H_2_O. Nutrient solutions were replaced every 4 days, and box positions were randomized to ensure uniform light exposure.

**Table 1:** The table details the nutrient compositions and adjustments made for LN (0.2 mM nitrogen) and NN (1 mM nitrogen) treatments.

Components	Quantity	DW in mL	Capacity	Weight in (g)	Final Concentration	Concentration (mmol/L)	1 m^3^ (g)	Concentration (mmol/L)	1 m^3^(g)
Ca(NO_3_)_2_.4H_2_O	2M	845	2000x	399.13	236.15	1	236.15	0.2	47.23
(NH_4_)_2_SO_4_	2M	1000	2000	264.28	132.14	1	132.14	0.2	26.428
CaCl_2_	1.0916M	1000	1333x	121.148	110.98	1.5	166.47	2.4	266.352
CaCl_2_.4H2O	0.90838M			133.55	147.02	1.5	220.53	2.4	352.48
KCl	3M	600	2000x	149	74.55	1.5	111.825	1.5	111.825
KH_2_PO_4_	0.4M	400		272	136.09	0.2	27.218	0.2	27.218
MgSO_4_.7H_2_O	2M	500	4000x	246.48	246.3	0.5	123.15	0.5	123.15
H_3_BO_3_	0.004M	500	4000x	0.124	61.83	0.001	0.062	0.001	0.062
Na_2_MoO_4_.2H_2_O				0.169	241.95	0.00035	0.085	0.00005	0.012
CuSO_4_.5H_2_O				0.25	249.68	0.0005	0.125	0.0005	0.125
ZnSO_4_.7H_2_O				0.575	287.54	0.001	0.288	0.001	0.288
MnCl_2_.4H_2_O				0.396	197.91	0.001	0.198	0.001	0.198
FeEDTA	0.2M	1000	2000x	73.43	367	0.1	36.7	0.1	36.7

Calcium concentrations were modified to 2.4 mM (LN) and 1.5 mM (NN) using CaCl_2_·4H_2_O. Solutions were replaced every 4 days to maintain consistency, ensuring uniform nutrient availability during the experiments conducted in controlled growth chamber conditions.

### Experimental design and replication

The study followed a completely randomized design with four independent biological experiments (one per seasonal cycle). Each landrace was assigned to three biological replicates per treatment (LN/NN) per experiment, totaling 24 plants per landrace (12 LN, 12 NN) across all experiments. Randomization of pot placement and treatment assignment was performed using a random number generator. Preliminary statistical analysis (one-way ANOVA, p < 0.05) confirmed no significant differences (p > 0.05) in measured traits between the four experiments, validating homogeneity of variance. Data from all experiments were pooled to enhance statistical robustness (total n = 3696 plants per treatment).

### Sampling and measurements

After 28 days of transplantation, the plants were harvested. At harvest, fresh plants were divided into shoot and root portions. The phenotyping process consisted of three main steps. First, for fresh weight measurements, the samples were blotted dry with paper towels to remove surface water and then weighed using an electronic balance. Second, shoot and root lengths were measured using a ruler. Third, for dry weight recording, the samples were dried in an oven at 70°C for 96 hours and then weighed. Overall, 23 root-shoot biomass traits were measured. These included traits: shoot length (SL), root length (RL), fresh shoot weight (FSW), fresh root weight (FRW), dry shoot weight (DSW), dry root weight (DRW), total biomass (TBM), and total dry matter (TDM); and 15 indirect traits: total moisture content (TMC), root-shoot length ratio (RSLR), fresh root-shoot weight ratio (FRSWR), fresh root biomass allocation efficiency (FRBAE), dry shoot biomass allocation efficiency (DSBAE) as major traits. root-shoot biomass traits in Watkins wheat landraces were quantified using the universal and modified formulas proposed by (Beadle et al., 1985; Kotowski et al., 2001; Bolinder et al., 2002; Belachew et al., 2019), providing a robust framework for biomass assessment in this diverse germplasm collection. The main phenotypic traits were calculated are available in **Supplementary Table S2**.

### Genome-wide association study and gene identification

The landrace data of the 308 Watkins wheat landraces used in this study is the same as the landrace data previously published by our team in Nature (Cheng et al., 2024). GWAS was carried out to detect marker-trait associations (MTAs) using the package MLM in R (Jiang et al., 2019). We considered −log_10_ (*P*-value) ≥ 5.0 (*P* ≤ 0.001) as the significance threshold. All SNPs that met the above cut-off value were identified as a total of 74,111 significant SNPs. MTAs After filtering, a total of 53,361 high-quality SNP markers (MAF > 0.05, missing rate per site< 10%) were screened out and were further utilized to perform GWAS for 23 seedling biomass-related traits. The GWAS results were visualized using Manhattan plots by the Genome Association and Prediction Integrated Tool (GAPIT) package (Wang et al., 2022). In the Manhattan plot, the x-axis and y-axis represent the chromosomal positions of SNPs and the −log_10_ (*P*-value) is derived from the F-test, respectively. The gene names and functions were identified on the International Wheat Genome Sequencing Consortium (IWGSC) RefSeq v1.0 website (https://wheat-urgi.versailles.inra.fr/), accessed on 24 December 2024. Genes involved in root-shoot biomass growth from other studies were considered putative candidate genes. The biological functions of the individual genes were obtained from UniProt (https://www.uniprot.org) and https://knetminer.rothamsted.ac.uk/. The physical map positions of all significantly associated SNPs were used to search and identify candidate genes in the http://wheat.cau.edu.cn/wGRN/ and http://wheat.cau.edu.cn/TGT/. The validation of the gene was confirmed using http://netminer.rothamsted.ac.uk/. The heatmap was created using TBtools software, and the haplotype plots, radial bar, and half violin bar were created using Origin 2021 software.

## Results

### Phenotypic variation in root-shoot-ground biomass

**Figure 1:**
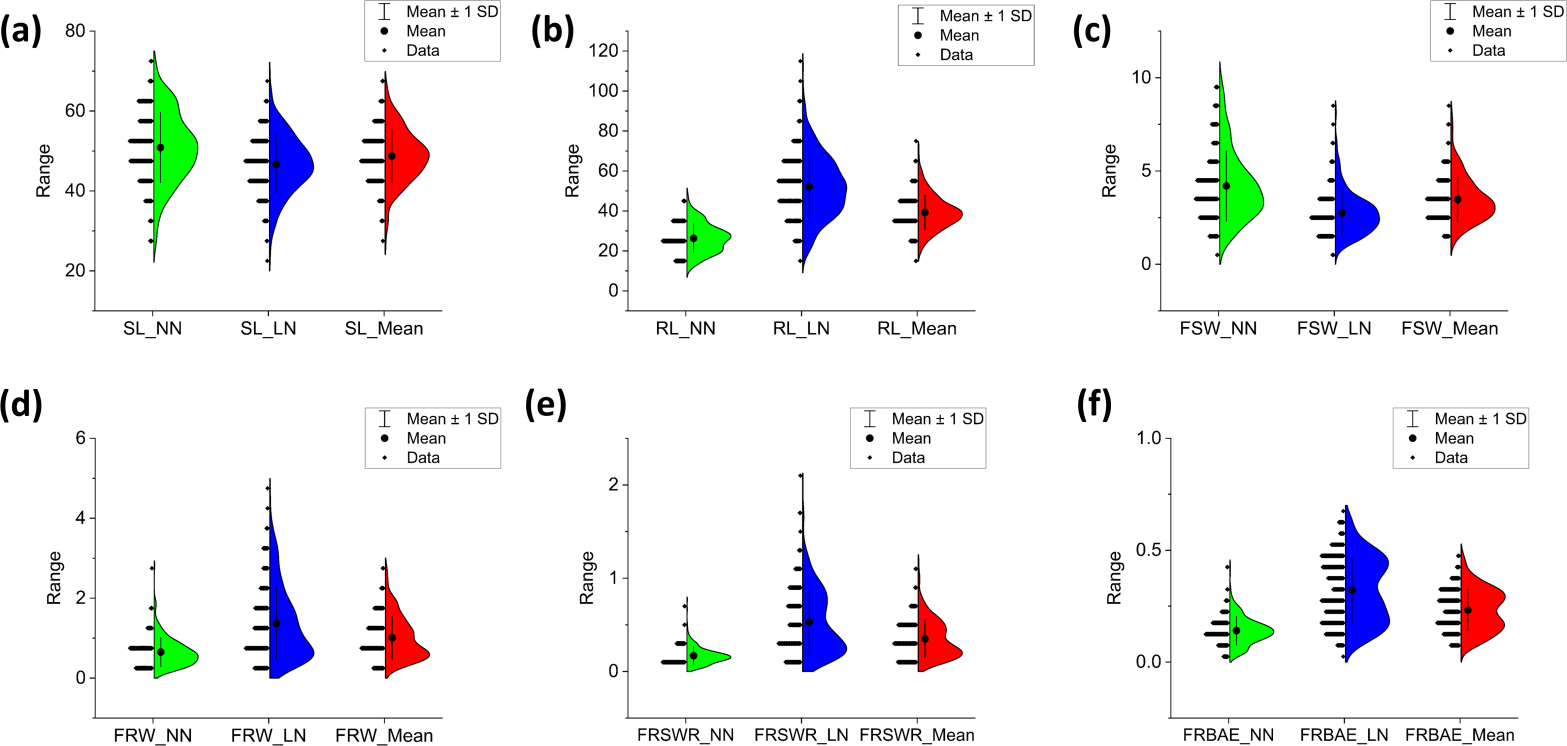
Phenotypic variation in root and shoot biomass-related traits across 308 Watkins wheat accessions. Half-violin plots display the distribution of six key traits: **(a)** shoot length (SL, cm), **(b)** root length (RL, cm), **(c)** fresh shoot weight (FSW, G), **(d)** fresh root weight (FRW, G), **(e)** root-to-shoot weight ratio (FRSWR), and **(f)** root biomass allocation efficiency (FRBAE). Each plot illustrates the data distribution (kernel density estimation), mean value (solid line), and variability (± 1 standard deviation, dashed lines). The traits were measured under controlled conditions, with length recorded in centimeters (cm) and weight in grams **(g)**. The plots highlight the broad phenotypic diversity within the Watkins wheat collection, providing insights into trait correlations and potential breeding targets.

Phenotypic variation in root-shoot biomass under varying nitrogen availability was assessed across 23 seedling traits. SL was slightly lower in LN (max: 69.6 cm, min: 22.4 cm) compared to NN (max: 73.3 cm, min: 26.7 cm), with landraces ranging from 45–55 cm in NN and 46–48 cm in LN (**Figure 1a**). RL increased more in LN (max: 112.8 cm, min: 17.9 cm) than in NN (max: 48.5 cm, min: 10.5 cm), with landraces ranging from 23–25 cm in NN and 50–60 cm in LN (**Figure 1b**). FSW was higher in NN (max: 9.95 g, min: 0.57 g) than LN (max: 6.97 g, min: 0.58 g), with most landraces showing a decrease in LN (**Figure 1c**). In contrast, FRW, FRSWR, and FRBAE were higher in LN, with FRW (max: 4.71 g, min: 0.17 g), FRSWR (max: 1.73, min: 0.02), and FRBAE (max: 0.801 g, min: 0.668 g) all showing increased values under LN (**Figures 1d-f**). Landrace ranges for these traits were also higher in LN. The response in both LN and NN treatment on each of the 308 Watkins wheat landraces for all traits is comprehensively documented in **Supplementary Table S1**, providing a detailed overview of the genetic diversity and phenotypic variability across the entire collection.

### Correlation analysis of phenotypic traits for root-shoot biomass

**Figure 2:**
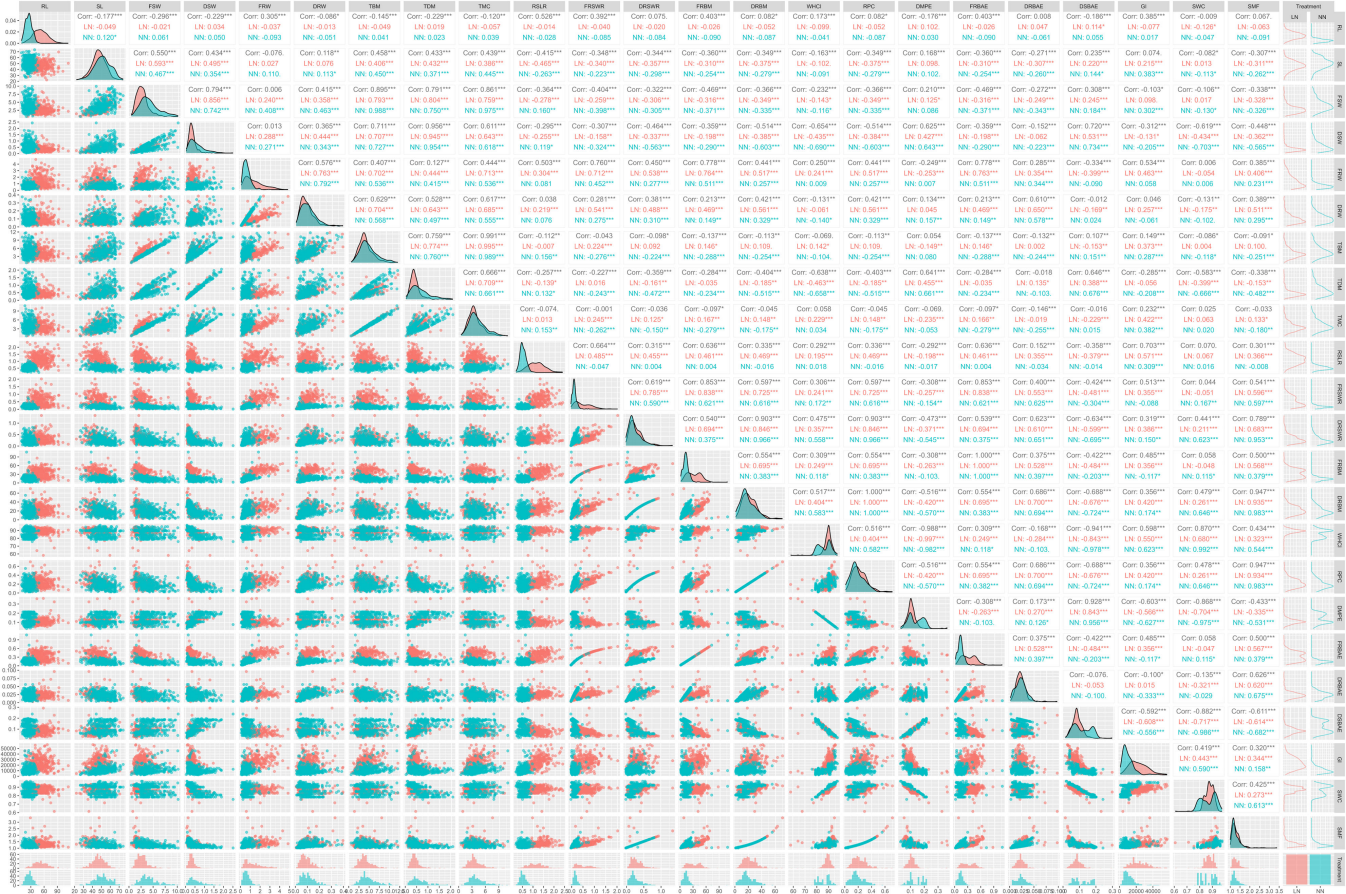
Correlation analysis between total biomass (TBM) and related traits across low nitrogen (LN) and normal nitrogen (NN) conditions. This figure illustrates the complex relationships between TBM and key traits, highlighting both positive and negative correlations. TBM shows strong positive correlations with shoot length (SL, 0.458***), fresh shoot weight (FSW, 0.895***), dry shoot weight (DSW, 0.711***), total dry matter (TDM, 0.759***), and total mineral content (TMC, 0.666***), consistently significant across both LN and NN conditions. Conversely, TBM is negatively correlated with root-to-shoot length ratio (RSLR, -0.257***), fresh root biomass (FRBM, -0.137***), dry root biomass (DRBM, -0.113**), and shoot mass fraction (SMF, -0.091**), though the strength and direction of these correlations vary between LN and NN. For example, FRBM exhibits a positive correlation with TBM under LN conditions (0.146*), whereas NN conditions reveal a negative correlation (-0.288***). Similarly, fresh root-to-shoot weight ratio (FRSWR) and dry root-to-shoot weight ratio (DRSWR) show divergent trends, with no significant overall correlation with TBM but notable differences between LN and NN. These findings underscore the importance of nitrogen availability in modulating the relationships between TBM and other traits. Statistical significance is denoted by asterisks: ***p < 0.001, **p < 0.01, *p < 0.05.icant correlations are marked with asterisks (***p < 0.001, **p < 0.01, *p < 0.05).

To investigate the phenotypic traits that may determine the yield of aboveground and belowground biomass, Pearson correlation analysis was conducted on twenty-eight-day-old plants. **Figure 2** provides detailed information about the correlations. TBM showed strong positive correlations with shoot-related traits, including SL (0.458), LN (0.406), and NN (0.450), emphasizing shoot architecture as a key productivity driver. The exceptionally high correlation with FSW (0.895) and DSW (0.711) underscores shoot biomass as the primary contributor to TBM. Positive associations with root traits like FRW (0.407) and DRW (0.629) suggest secondary contributions from root development. Strong linkages with TDM (0.759) and TMC (0.666***) highlight the importance of structural and hydraulic partitioning. Consistency across LN and NN genotypes implies conserved shoot-driven biomass mechanisms. TBM correlated negatively with root specialization traits, including RSLR (-0.257**), suggesting that shoot prioritization enhances yield. Genotype-specific patterns emerged: LN showed weaker negative correlations (-0.139), while NN paradoxically exhibited positive trends (0.132*), reflecting genetic variation in allocation strategies. Similarly, FRSWR and DRSWR diverged between LN (positive) and NN (negative), highlighting opportunities for genotype-specific root architecture breeding. Negative correlations with root biomass allocation (FRBM: -0.137; DRBM: -0.113) and water-related traits (WHC: -0.069; SWC: -0.086) suggest excessive root investment or water retention limits yield. Inverse relationships with root physiology (RPC: -0.113; FRBAE/DRBAE: -0.137* to -0.132) indicate metabolic trade-offs between root function and whole-plant growth. The weak negative correlation with SMF (-0.091) further supports dry matter over hydration as a TBM driver. TBM correlated positively with GI (0.149*) and DSBAE (0.107), emphasizing growth vigor and shoot carbon allocation. While DMPE showed no overall link, contrasting LN (-0.149**) and NN (0.080) trends suggest genotype-specific metabolic efficiency. NN prioritized shoot biomass, whereas LN balanced allocation, reflecting divergent strategies.

### Relationship among traits through principal component analysis

**Figure 3:**
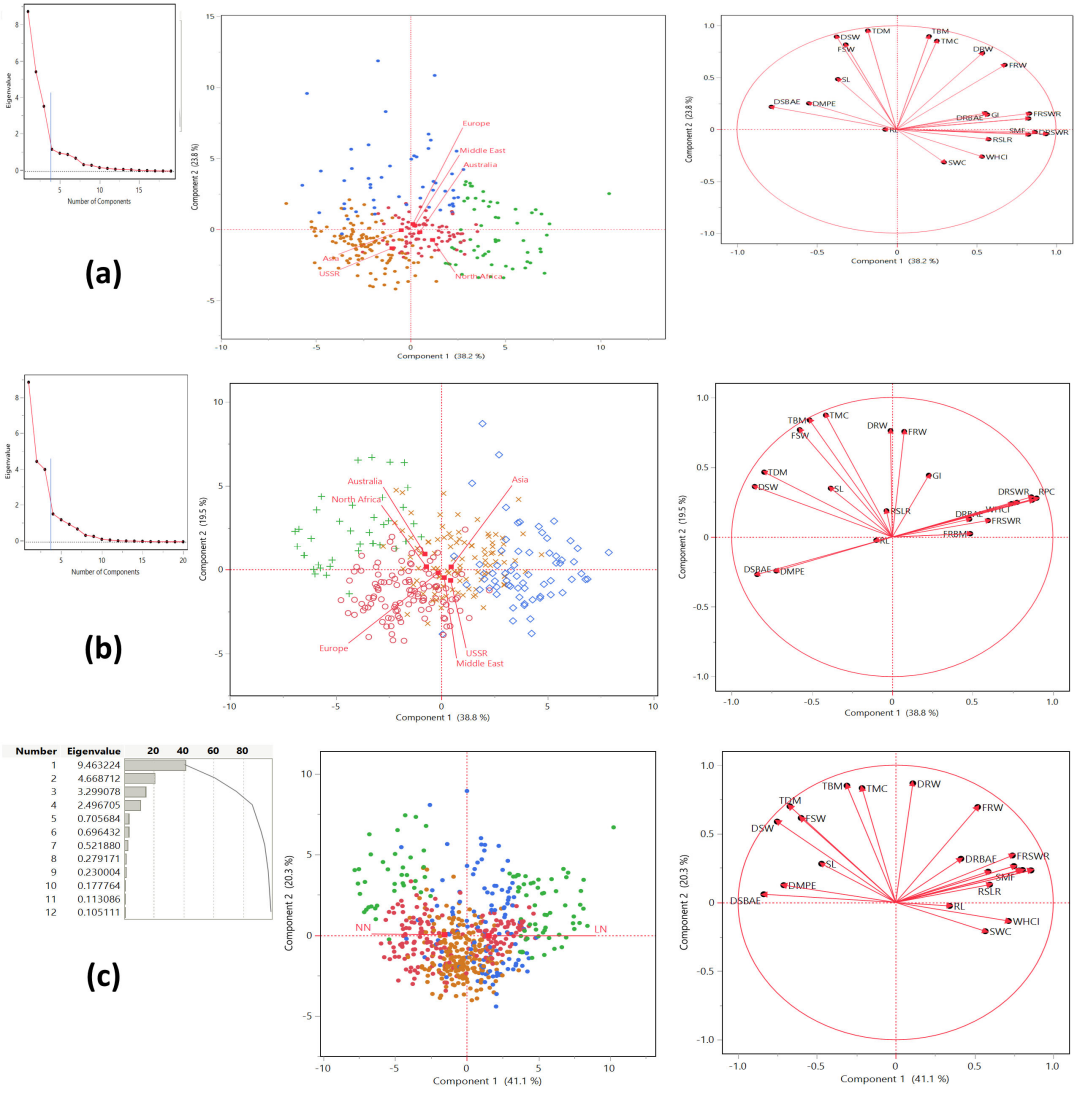
Principal Component Analysis (PCA) of trait correlations and geographic distribution under low nitrogen (LN) and normal nitrogen (NN) treatments. **(a)** Low nitrogen (LN) treatment: The PCA plot highlights the significant contributions of variables to Principal Component 1 (PC1), with landraces from Europe, the Middle East, Australia, and North Africa predominantly aligning with PC1, while those from Asia and the USSR align more with Principal Component 2 (PC2). **(b)** Normal nitrogen (NN) treatment: Under NN conditions, variable contributions shift, with landraces from Asia, the USSR, and the Middle East aligning with PC1, whereas those from Australia, North Africa, and Europe align with PC2. **(c)** Variance explained by the first four principal components: PC1 explains 41.144% of the total variance (eigenvalue = 9.463), PC2 accounts for 20.299% (eigenvalue = 4.669), PC3 for 14.344% (eigenvalue = 3.299), and PC4 for 10.855% (eigenvalue = 2.497), collectively capturing 86.642% of the total variance. All components are statistically significant (p < 0.0001). The PCA reveals clear patterns of trait correlations and geographic distribution under contrasting nitrogen treatments, emphasizing how nitrogen availability influences both the relationships among traits and the clustering of landraces from different geographic regions.

The principal component analysis reveals distinct patterns of correlation among the traits across the first four principal components. The landraces in this study were classified into four distinct clusters based on their performance under LN and NN conditions. Each cluster contained landraces from diverse geographic regions, including Asia, Australia, Europe, the Middle East, North Africa, and the USSR. Under LN conditions, Cluster 1 was predominantly represented by landraces from Europe (29) and Asia (20), while Cluster 4 had the highest number of landraces from Asia (51) and Europe (32). Similarly, under NN conditions, Cluster 1 was dominated by landraces from Europe (46) and Asia (29), and Cluster 4 included significant contributions from Asia (34) and Europe (28) (**Figures 3a, b**; **Table 2**). This distribution highlights the genetic diversity and adaptability of landraces across different regions, as they were grouped into clusters based on their response to nitrogen availability. The presence of landraces from multiple regions in each cluster suggests that geographic origin does not strictly determine performance under varying nitrogen conditions, emphasizing the importance of genetic factors in adaptation. The first component explained the largest portion of the variance, accounting for 41.144% with an eigenvalue of 9.463224, and was highly significant (chi-square = 30699.3, p < 0.0001). The second component explained 20.299% of the variance, with an eigenvalue of 4.668712, and was also highly significant (chi-square = 25546). The third component captured 14.344% of the variance, with an eigenvalue of 3.299078, and was significant (chi-square = 21954.6). The fourth component explained 10.855% of the variance, with an eigenvalue of 2.496705, and was significant (chi-square = 18131.9). Together, the first four components explained 86.642% of the total variance, indicating that they capture the majority of the information in the dataset. All components were statistically significant (p < 0.0001), confirming that they represent meaningful patterns in the data (**Figure 3c**). In Principal Component 1 (Prin1), traits such as DRBM (0.858792), DRSWR (0.805009), SMF (0.782583), FRBAE (0.7499), FRSWR (0.741075), WHCI (0.716023), RSLR (0.596359), GI (0.58677), FRW (0.52041), SWC (0.568605), DRBAE (0.41438), and RL (0.342267) exhibit strong positive correlations with each other, indicating that they share common underlying variance. Conversely, traits like DSBAE (-0.837217), DSW (-0.752274), DMPE (-0.710679), TBM (-0.309508), TMC (-0.213536), TDM (-0.671679), FSW (-0.600394), and SL (-0.470496) show strong negative correlations with these traits, suggesting an inverse relationship. Prin1 effectively captures a contrast between these two groups of traits. Moving to Principal Component 2 (Prin2), traits such as DRW (0.867133), TBM (0.850217), TMC (0.832145), TDM (0.699745), FRW (0.694469), and FSW (0.615037) are positively correlated, while WHCI (-0.135041) and SWC (-0.209736) are negatively correlated with them, highlighting another dimension of variability. In Principal Component 3 (Prin3), DSBAE (0.50825), DMPE (0.654167), and DRBAE (0.572137) are positively correlated, whereas WHCI (-0.649644), SWC (-0.724641), TBM (-0.395442), TMC (-0.477095), FSW (-0.45137), and SL (-0.386778) are negatively correlated, indicating a distinct separation between these groups. Finally, in Principal Component 4 (Prin4), traits like FRBAE (0.346615), FRSWR (0.287942), RSLR (0.552824), GI (0.505017), and FRW (0.341096) are positively correlated, while DRBM (-0.427753), DRSWR (-0.374719), SMF (-0.428867), DRW (-0.324427), and DRBAE (-0.537776) are negatively correlated, further emphasizing the contrasting relationships between these traits. Together, these principal components provide a comprehensive view of the underlying structure and correlations among the traits, with specific loadings quantifying the strength and direction of these relationships.

**Table 2:** The table presents the distribution of landraces from different geographic regions (Asia, Australia, Europe, Middle East, North Africa, and USSR) across four clusters under both low nitrogen (LN) and normal nitrogen (NN) conditions.

LN					Total	NN				Total	Per%
Row Labels	1	2	3	4		1	2	3	4		
Asia	20	19	12	51	102	29	11	28	34	102	33.12
Australia	11	3	4	7	25	7	2	2	14	25	8.12
Europe	29	19	24	32	104	46	13	17	28	104	33.77
Middle East	10	8	8	12	38	14	4	9	11	38	12.34
North Africa	4	10	2	9	25	6	7	4	8	25	8.12
USSR	3	1		10	14	7		5	2	14	4.55
Grand Total	77	60	50	121	308	109	37	65	97	308	

The columns show the number of landraces in each cluster (1 to 4) and the total number of landraces per region for both LN and NN treatments. The grand total at the bottom summarizes the overall distribution, with 308 landraces analyzed. This table highlights the regional diversity and clustering patterns of landraces under varying nitrogen conditions, providing insights into their adaptability and performance.

### Relationship among traits through hierarchical cluster analysis

**Figure 4:**
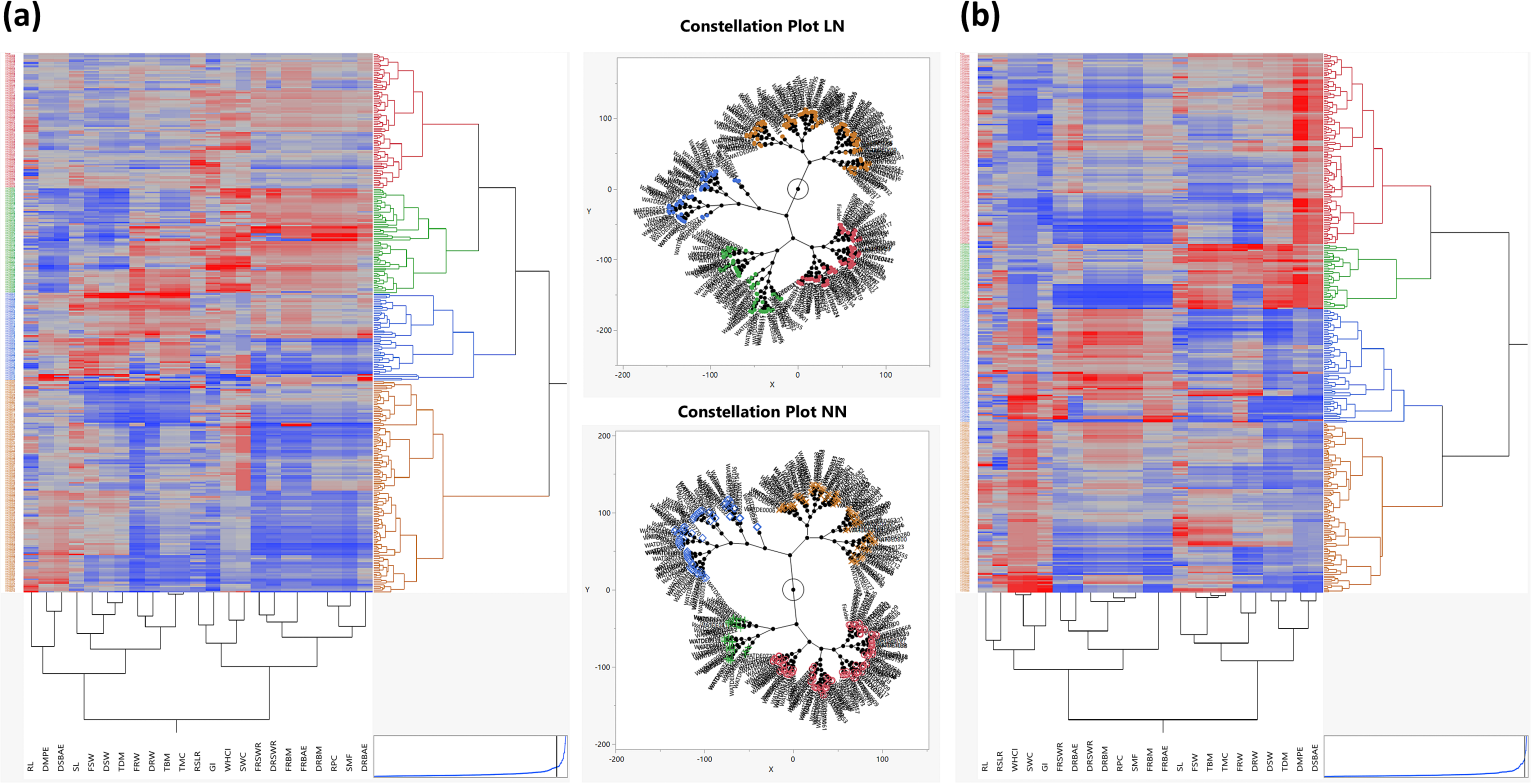
Clustering analysis of landraces using agglomerative hierarchical clustering (ahc) and scatter plot matrices under low nitrogen (LN) and normal nitrogen (NN) conditions. **(a)** Low nitrogen (LN): A dendrogram generated using AHC with Ward’s method, based on the Euclidean distance matrix, groups landraces into four distinct clusters. The corresponding scatter plot matrix, constructed using factor scores from Principal Component 1 (PC1) and Principal Component 2 (PC2), reveals two main groups. One group, represented in yellow, shows no further subdivisions, while the second group includes three distinct subgroups, colored blue, green, and red. **(b)** Normal nitrogen (NN): The dendrogram and scatter plot matrix for NN conditions reveal similar clustering patterns. The constellation plot divides landraces into two main groups. Each main group contains two subgroups: one group consists of yellow and blue clusters, while the other comprises green and red clusters. The analysis highlights consistent clustering patterns across nitrogen treatments, with slight variations in subgroup distribution, reflecting the impact of nitrogen availability on trait-driven clustering of landraces.

To explore the genetic diversity and grouping patterns among the landraces, a scatter plot matrix was constructed using factor scores derived from PC1 and PC2. This matrix revealed four distinct clusters, indicating clear grouping patterns among the landraces. To further investigate these groupings, agglomerative hierarchical clustering (AHC) was employed using Ward’s method (Ward Jr, 1963), which calculated the Euclidean distance matrix and generated a dendrogram (**Figures 4a, b**). This method is widely recognized for its effectiveness in assessing genetic diversity within germplasm under contrasting environmental conditions. Additionally, a two-way clustering analysis was performed using the AHC method, resulting in a two-way cluster diagram and constellation plots. The constellation plot for the LN treatment was divided into two main groups. The first main group, represented in yellow, contained no subgroups, while the second main group consisted of three subgroups, represented in blue, green, and red. In the case of the NN constellation plot, it was also divided into two main groups, each containing two subgroups. One main group was represented in yellow and blue, while the other was represented in green and red. These visualizations provided further insights into the relationships among landraces. The primary aim of this study was to identify landraces demonstrating superior biomass production under both low and normal nitrogen conditions. To achieve this, we systematically screened and selected 50 landraces from FSW, FRW, and TBM in both LN and NN treatments. In our results, we looked insights into the performance of landraces across three key traits: FSW, FRW, and BM under LN conditions. The analysis reveals that Europe dominates in terms of the number of landraces, with 23 landraces in FSW, 20 in FRW, and 23 in TBM. This indicates that European landraces are well-represented and potentially well-adapted to low nitrogen environments. Asia follows with 10 landraces in each trait, while the Middle East contributes 7 landraces in FSW and TBM, and 4 in FRW. Australia and North Africa have fewer landraces, with 4 and 2 landraces respectively in FRW and TBM. The results also highlighted the performance of landraces under NN conditions across three key traits: FSW, FRW, and TBM. Europe and Asia dominate in terms of the number of landraces, with Europe contributing 13 landraces in FSW, 19 in FRW, and 18 in TBM, while Asia contributes 18 in FSW, 15 in FRW, and 16 in TBM. Other regions, such as Australia, North Africa, and the Middle East, also show significant representation, though with fewer landraces. The complete list of recommended landraces, based on their performance in each trait, is provided in **Supplementary Tables S3** and **S4**.

### Identification of significant SNPs associated with root-shoot biomass

**Figure 5:**
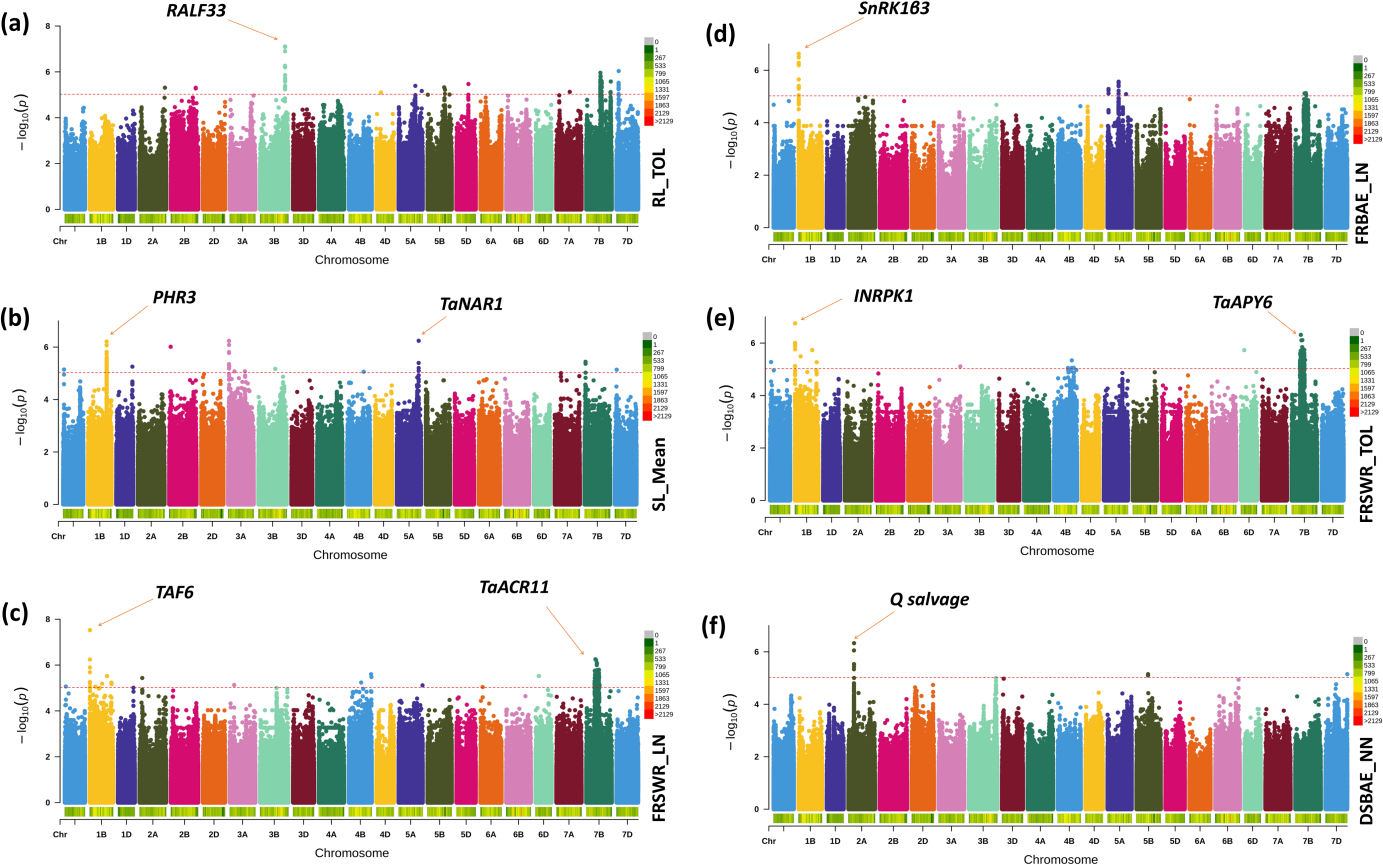
The Manhattan plot shows the significant SNPs across the Watkins Wheat Landraces chromosomes identified by the current GWAS analysis for root-shoot biomass. Genetic associations of SNPs with biomass-related traits in Watkins wheat: **(a)** SNP chr3B_735416309 on chr3B, associated with the gene *RALF33*, which encodes Protein RALF-like 33, plays a significant role in root length tolerance. **(b)** SNPs on chr5A (chr5A_639115931) and chr1B (chr1B_544451013), linked to the genes *TaNAR1* and *PHR3*, respectively, are associated with increased above-ground biomass. **(c)** SNPs chr1B_4329347 on chr1B and chr7B_270480487 on chr7B, linked to the genes *TAF6* and *TaACR11*, respectively, are associated with fresh root-shoot weight ratio under low nitrogen conditions. **(d)** SNP chr1B_3576292 on chr1B, associated with the gene *SnRK1β3*, is linked to root biomass allocation under low nitrogen conditions. **(e)** SNP chr7B_270480487 on chr7B, associated with the gene *TaAPY6*, and SNP chr1B_4329347 on chr1B, associated with *INRPK1*, are linked to fresh root-shoot weight ratio tolerance. **(f)** SNP chr2A_164500047, associated with the gene *Q salvage*, is involved in dry shoot biomass allocation.

Our results identified several key SNPs, linked to specific genes, that are significantly associated with root-shoot biomass traits in Watkins wheat. The list of all SNPs has been included in **Supplementary Table S5**. We have included the description/annotation and function of characterized genes to control different root-shoot biomass traits in **Supplementary Table S6**. We only discussed key genes summarized in **Table 3**. These SNPs, their p-values, and candidate gene. On chr3B, the SNP chr3B_735416309 has a significant effect of 7.266* and a p-value of 7.11, associated with the gene *RALF33* in root length tolerance (**Figure 5a**). Our findings also highlight that on chr5A, the SNP chr5A_639115931 has an effect of 4.110* and a p-value of 6.24, linked to the gene *TaNAR1*. On chr1B, the SNP chr1B_544451013 has an effect of 3.791* and a p-value of 6.24, associated with the gene *PHR3.* These genes play a major role in increasing above-ground biomass (**Figure 5b**). Additionally, we identified two SNPs: chr1B_4329347 on chr1B, which has a negative effect of -0.128* and a p-value of 7.53, associated with the gene *TAF6* and chr7B_270480487 on chr7B, which has a positive effect of 0.131* and a p-value of 6.25, linked to the gene *TaACR11*. Both are associated with the fresh root shoot weight ratio under low nitrogen conditions (**Figure 5c**). This study also revealed that on chr1B, the SNP chr1B_3576292 has a negative effect of -0.050* and a p-value of 6.63, linked to the gene *SnRK1β3*. It is associated with root biomass allocation under low nitrogen conditions (**Figure 5d**). Interestingly, the SNP chr7B_270480487 also has a negative effect of -0.132* and a p-value of 6.31, associated with the gene *TaAPY6*. On chr1B, the SNP chr1B_4329347 has a positive effect of 0.121* and a p-value of 6.75, linked to the gene *INRPK1*, associated with fresh root shoot weight ratio tolerance (**Figure 5e**). Finally, we found that the SNP chr2A_164500047, with an effect of 0.031* and a p-value of 6.33, is associated with the gene *Q salvage* for dry shoot biomass allocation (**Figure 5f**). These results provide valuable insights into the genetic architecture of biomass-related traits in Watkins wheat, highlighting key SNPs and their associated genes that may play crucial roles in regulating root-shoot biomass.

**Table 3:** The table lists significant single nucleotide polymorphisms (SNPs) identified in Watkins wheat, along with their genomic positions, p-values, associated gene names, gene codes, and the corresponding traits related to root-shoot biomass.

SNP	Position	p value	Gene Name	Gene code	Trait	Reference
chr6B_615645229	615645229	6.551277068	*OHP1*	*TraesCS6B02G454500*	DMPE_GMP	(Curtis et al., 2019)
chr2A_164500047	164500047	6.329161536	*Q salvage*	*TraesCS2A02G194400*	DSBAE_NN	(Xu et al., 2015)
chr1B_3576292	3576292	6.631110531	*SnRK1β3*	*TraesCS1B02G005600*	FRBAE_LN	(Margalha et al., 2019)
chr1B_4329347	4329347	7.525196792	*TAF6*	*TraesCS1B02G007800*	FRSWR_LN	(Parvathi and Nataraja, 2017; Li et al., 2023)
chr7B_270480487	270480487	6.253318017	*TaACR11*	*TraesCS7B02G186500*	FRSWR_LN	(Ma, 2010)
chr1B_4329347	4329347	6.751401593	*INRPK1*	*TraesCS1B02G007400*	FRSWR_TOL	(Bassett et al., 2000)
chr7B_270480487	270480487	6.312880191	*TaAPY6*	*TraesCS7B02G178800*	FRSWR_TOL	(Chowdhury et al., 2023)
chr3B_735416309	735416309	7.10783438	*RALF33*	*TraesCS3B02G488900*	RL_TOL	(Xue et al., 2024)
chr5A_639115931	639115931	6.240206033	*TaNAR1*	*TraesCS5A02G047900*	SL_Mean	(Zhao et al., 2013)
chr1B_544451013	544451013	6.236231896	*PHR3*	*TraesCS1B02G320000*	SL_Mean	(Zheng et al., 2020)

Key genes such as *OHP1*, *SnRK1β3*, *TaACR11*, and *PHR3* are highlighted, along with their roles in traits like DMPE_GMP, DSBAE_NN, FRBAE_LN, FRSWR_LN, FRSWR_TOL, RL_TOL, and SL_Mean. This table provides insights into the genetic markers and candidate genes influencing biomass traits under varying conditions, offering valuable information for future breeding and genetic studies.

### Genomic characterization and functional analysis of candidate genes

**Figure 6:**
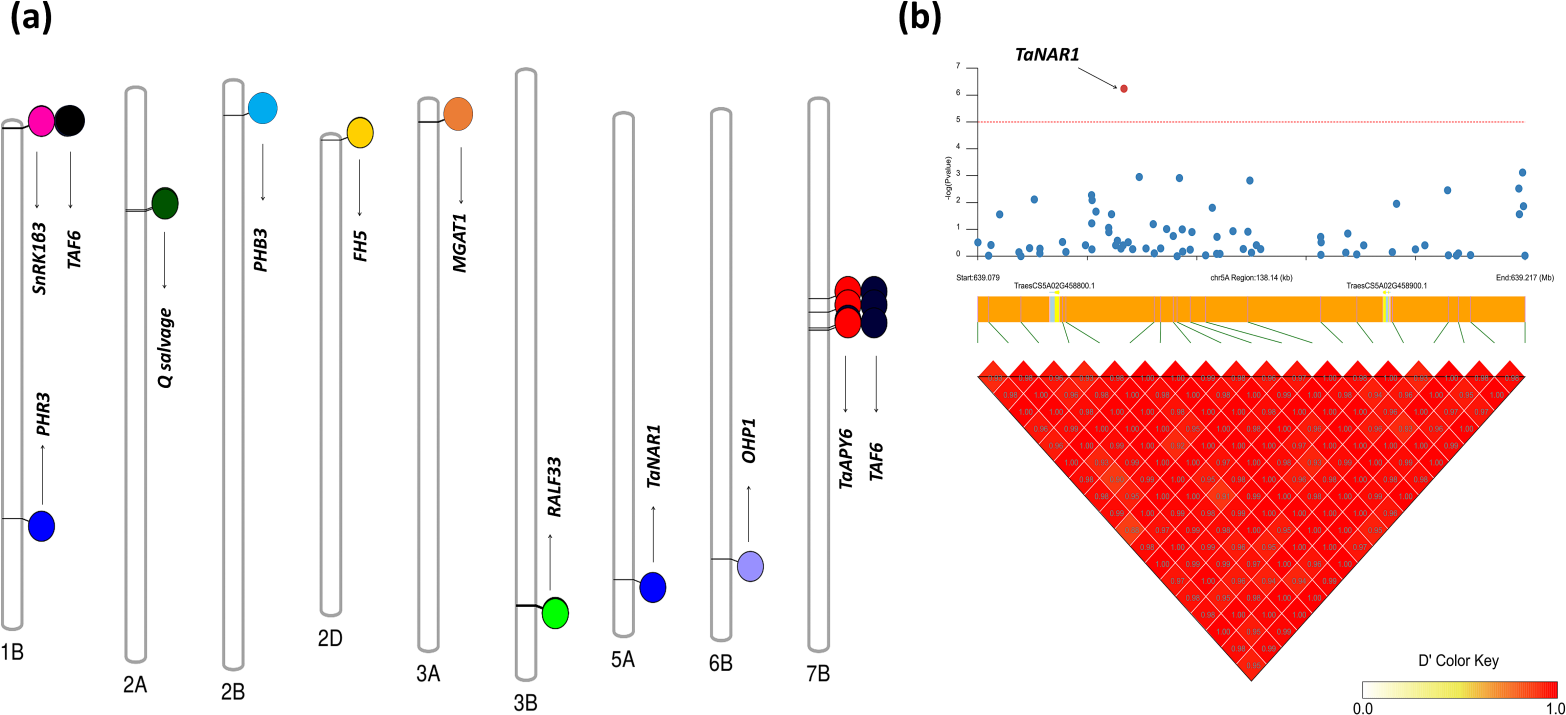
Chromosome map and linkage disequilibrium (LD) analysis of genes associated with root-shoot biomass traits in Watkins Wheat Landraces. **(a)** Chromosome map of selected genes: The map highlights the locations of genes associated with biomass-related traits. Three genes are located on Chr1B: *PHR3*, *SnRK1β3*, and *TAF6*, linked to shoot length, fresh root biomass, and fresh root-to-shoot weight ratio, respectively. The gene *Q salvage* on Chr2A controls the dry shoot biomass trait, while *PHB3* on Chr2B is associated with the dry root-to-shoot biomass ratio. The gene *FH5* on Chr2D regulates total dry matter. On Chr3A, *MGAT1* is associated with the root-to-shoot ratio, whereas RALF33 on Chr3B plays a significant role in root length. *TaNAR1* on Chr5A is linked to shoot length, while OHP1 on Chr6B contributes to dry matter production. Finally, *TaAPY6* and *TAF6* are found at three distinct positions on Chr7B and are associated with the fresh root-to-shoot weight ratio. **(b)** Linkage disequilibrium (LD) analysis: The LD analysis confirms the position of the candidate gene *TaNAR1* (*TraesCS5A02G458800*) on Chr5A, demonstrating its role in regulating above-ground biomass growth. This figure provides a comprehensive overview of the genomic regions and candidate genes underlying key root-shoot biomass traits, supported by LD analysis that validates the positional accuracy of significant genes.

Our study revealed significant insights into the genomic characterization and functional roles of candidate genes associated with root-shoot biomass traits in Watkins wheat landraces. We identified key SNPs significantly linked to these traits, as illustrated in **Figure 6a**, which represents a chromosome map showing the actual locations of selected genes. Furthermore, linkage disequilibrium (LD) analysis, as shown in **Figure 6b**, confirmed the position of the candidate gene *TaNAR1* (*TraesCS5A02G458800*) at chr5A_639115931, which is involved in regulating above-ground biomass growth.

### Functional analysis of candidate genes

**Figure 7:**
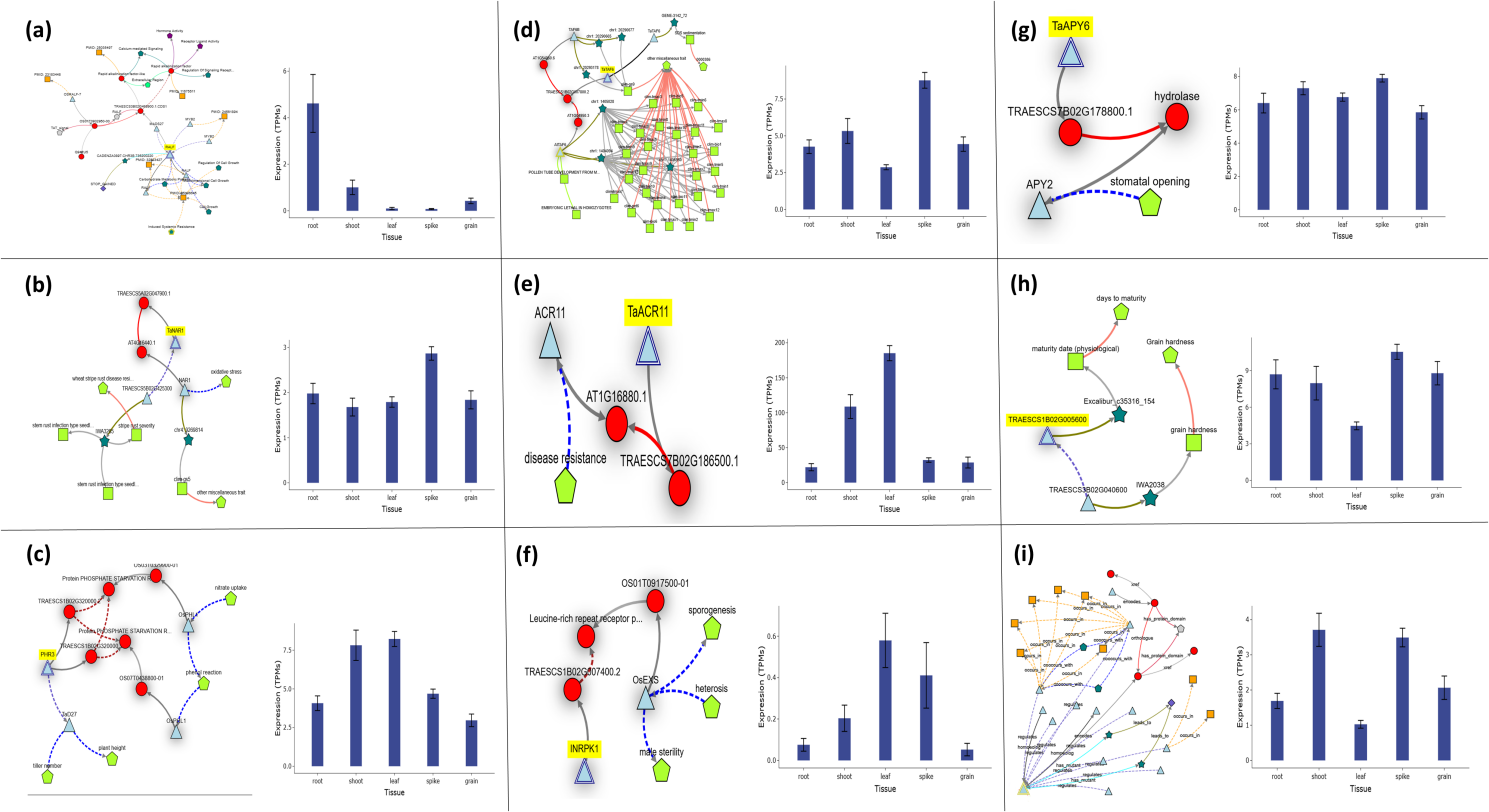
Expression and key functions of individual candidate genes across Watkins Wheat Landraces chromosomes, as identified by the current GWAS analysis for root-shoot biomass traits. **(a)** The gene *RALF33* on chr3B (chr3B_735416309), encoding Protein RALF-like 33, plays a significant role in controlling root biomass. *RALF33* is a member of the RALF family and is involved in cell growth, metabolic processes, and signaling regulation. **(b)** *TaNAR1* on chr5A (chr5A_639115931), encoding Protein NAR1, is linked to the regulation of root-shoot biomass. *TaNAR1* is associated with oxidative stress regulation and stem growth in wheat at the seedling stage. **(c)** *PHR3* on chr1B (chr1B_544451013), encoding the Myb family transcription factor PHL7, is associated with the regulation of leaf and stem biomass. *PHR3* is involved in regulating plant height, tiller number, and nitrate uptake. **(d)** The gene *TAF6* on chr1B (chr1B_4329347), encoding the transcription initiation factor TFIID subunit 6, plays an important role in controlling spike biomass. *TAF6* is also involved in SDS sedimentation, pollen tube development, and embryonic lethality in homozygotes. **(e)** *TaACR11* on chr7B (chr7B_270480487), encoding the ACT domain-containing protein DS12 (chloroplastic), is involved in regulating leaf biomass. *TaACR11* is associated with disease resistance regulation. **(f)** *INRPK1* on chr1B (chr1B_4329347), encoding the probable leucine-rich repeat receptor-like protein kinase At1g35710, regulates leaf and spike biomass. *INRPK1* is involved in stomatal opening and hydrolase activities. **(g)** *TaAPY6* on chr7B (chr7B_270480487), encoding probable apyrase 6, plays a significant role in controlling both root-shoot biomass. *TaAPY6* is associated with maturity and grain hardness regulation. **(h)** The gene *SnRK1β3* on chr1B (chr1B_3576292), encoding the SNF1-related protein kinase regulatory subunit beta-3, plays a significant role in controlling both root-shoot biomass. *SnRK1β3* is involved in sporogenesis, male sterility, and heterosis. **(i)** Finally, the gene *Q salvage* on chr2A (chr2A_164500047), encoding the queuosine salvage protein, is associated with the regulation of stem and spike biomass. *Q salvage* is involved in defense responses and secretion regulation.

Functional analysis of candidate genes unveiled a complex network of regulatory mechanisms influencing biomass traits in plants, highlighting their diverse roles in both developmental and stress-responsive pathways. The gene *RALF33*, located on chromosome 3B, encodes RALF-like 33, a protein implicated in processes such as cell growth, metabolism, and signaling, ultimately influencing below-ground biomass, as illustrated by its phenotypic and expression profiles (**Figure 7a**). Contrasting this below-ground focus, *TaNAR1* (chromosome 5A) emerged as a critical regulator of above-ground biomass, while also governing oxidative stress responses and conferring resistance to stem rust pathogens, underscoring its dual role in growth and defense (**Figure 7b**). Meanwhile, *PHR3* (chromosome 1B), a Myb transcription factor, demonstrated a broader influence on plant architecture, modulating traits such as plant height, tiller number, and nitrate uptake efficiency, all of which collectively contribute to above-ground biomass accumulation (**Figure 7c**). Another gene on chromosome 1B, *TAF6*, a subunit of the TFIID complex, revealed unexpected pleiotropy, linking its role in transcription initiation to diverse phenotypes including SDS sedimentation—a marker of gluten quality—pollen tube development, and embryonic lethality, suggesting its fundamental importance across developmental stages (**Figure 7d**). Further expanding the functional spectrum, *TaACR11* (chromosome 7B), an ACT domain-containing protein, bridged the gap between disease resistance and biomass regulation, illustrating how metabolic signaling pathways might integrate stress adaptation with growth (**Figure 7e**). Similarly, *INRPK1*, a receptor-like kinase on chromosome 1B, influenced stomatal dynamics and hydrolase activity, processes critical for water-use efficiency and enzymatic regulation, which may indirectly shape biomass under fluctuating environmental conditions (**Figure 7f**). The apyrase-encoding gene *TaAPY6* (chromosome 7B) further highlighted the interconnectedness of growth and stress traits, impacting maturity timing, grain hardness, and both above- and below-ground biomass, suggesting its role in energy metabolism and resource allocation (**Figure 7g**). The *SnRK1β3* kinase subunit (chromosome 1B) added another layer of complexity, linking energy-sensing pathways to reproductive success through its effects on sporogenesis, male sterility, and heterosis—traits vital for yield optimization (**Figure 7h**). Finally, the *Q salvage* gene (chromosome 2A) tied defense responses and secretion mechanisms to above-ground biomass modulation, reinforcing the idea that growth-defense trade-offs are genetically embedded and dynamically regulated (**Figure 7i**).

### Gene expression of characterized and uncharacterized genes

**Figure 8:**
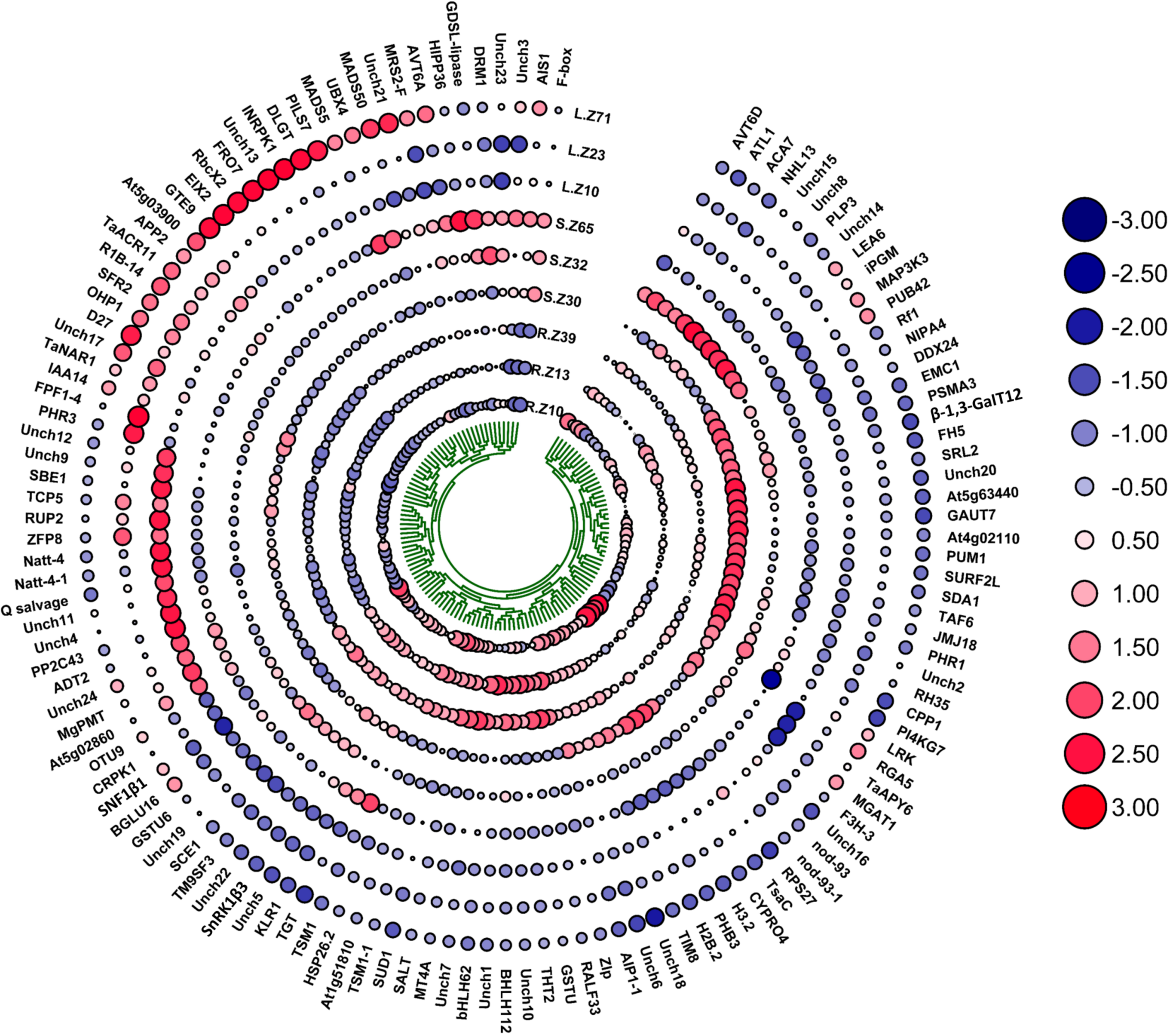
This heatmap illustrates the expression profiles of genes linked to biomass traits, with a color scale representing the direction of expression. Blue indicates downregulation (negative expression), while red signifies upregulation (positive expression). The size of each circle reflects the magnitude of gene expression: smaller circles correspond to lower expression levels, and larger circles represent higher expression levels. The expression levels are categorized into five groups, with progressively larger circles indicating greater expression intensity. Genes are evaluated for their contributions to both above-ground and below-ground biomass traits, offering insights into their regulatory roles in biomass development under varying environmental and nitrogen conditions. This visualization highlights the functional diversity and expression dynamics of key genes involved in biomass allocation, aiding in the identification of potential regulators for improving nitrogen use efficiency in wheat.

The current study identified 106 characterized and 24 uncharacterized candidate genes associated with multiple biomass-related traits in Watkins wheat, highlighting their potential roles in regulating root-shoot biomass development. **Table 4** and **Figure 8** summarize these genes and their associations with various traits. For the trait DMPE_GMP, our results revealed that gene *OHP1* exhibited the highest expression at 34.234 in l_Z10, 54.487 in l_Z23, and 59.068 in l_Z71. For DSBAE_NN, we found that gene *Q salvage* showed dominant expression at 4.122 in r_Z10, 2.144 in r_Z13, and 2.098 in r_Z39. In the trait FRBAE_LN, gene *SnRK1β3* exhibited the highest expression at 14.133 in r_Z10, 15.647 in r_Z13, and 16.469 in r_Z39. For FRSWR_LN, our study identified *TaACR11* as the gene with the highest expression, showing values of 18.098 in r_Z10, 12.946 in r_Z13, and 13.932 in r_Z39. Additionally, we found that gene *INRPK1* had the highest expression at 80.658 in r_Z10, 96.090 in r_Z13, and 88.164 in r_Z39 in FRSWR_TOL. In RL_TOL, gene *RALF33* demonstrated dominant expression at 2.445 in r_Z10, 6.118 in r_Z13, and 6.024 in r_Z39. Finally, our study revealed that gene *TaNAR1* showed the highest expression at 6.346 in r_Z10, 9.824 in r_Z13, and 6.919 in r_Z39, while gene *PHR3* also exhibited notable expression with values of 6.346 in r_Z10, 9.824 in r_Z13, and 6.919 in r_Z39 for SL_Mean. In this study, we identified 24 uncharacterized genes with high expression levels across various biomass-related traits, which may play significant roles in biomass regulation. The gene *Unch1* (*TraesCS1A02G32370*0) exhibited significant expression levels of 3.242 in r_Z13 and 1.938 in r_Z39 for DMPE_GMP. We noticed that gene *Unch2* (*TraesCS5D02G267700*) showed increased expression levels of 0.679 in r_Z13 and 0.689 in r_Z39 in DRBAE_GMP. For DRBAE_TOL, *Unch3* (*TraesCS1D02G156200*) displayed elevated expression at 4.486 in r_Z10 and 3.669 in r_Z39. In DRBM_TOL, gene *Unch6* (*TraesCS4A02G264600*) exhibited exceptionally high expression at 115.476 in r_Z10, 95.787 in r_Z13, and 90.718 in r_Z39. The gene *Unch10* (*TraesCS6B02G396300*) demonstrated high expression levels of 7.126 in r_Z13 and 6.15 in r_Z39 in DRSWR_NN. In DRW_TOL, gene *Unch11* (*TraesCS3D02G206900*) showed significant expression levels of 11.657 in r_Z10, 9.433 in r_Z13, and 10.577 in r_Z39. Our results also revealed that gene *Unch15* (*TraesCS4B02G186100*) exhibited the highest expression levels of 1.882 in r_Z13 and 2.084 in r_Z39 in FRSWR_TOL. In RL_TOL, gene *Unch18* (*TraesCS7B02G221300*) demonstrated consistent expression levels of 2.321 in r_Z13 and 2.35 in r_Z39. For RSLR_LN, the gene *Unch19* (*TraesCS2A02G481100*) showed elevated expression levels of 18.979 in r_Z13 and 15.754 in r_Z39. We found that gene *Unch20* (*TraesCS2A02G194900*) exhibited higher expression at 2.567 in r_Z13 and 2.108 in r_Z39 in SWC_NN. For TBM_Mean, the gene *Unch21* (*TraesCS6D02G166000*) demonstrated the highest expression levels at 3.683 in r_Z13 and 6.367 in r_Z39, while gene *Unch22* (*TraesCS7B02G247900*) also exhibited elevated expression at 3.071 in r_Z13 and 3.158 in r_Z39. Finally, the gene *Unch24* (*TraesCS2D02G190900*) exhibited significant expression at 0.529 in r_Z39 in TDM_NN.

**Table 4:** Description/Annotation and function of characterized/characterized genes and their to control different above and below ground biomass traits.

Gene Name	r_Z10	r_Z13	r_Z39	s_Z30	s_Z32	s_Z65	l_Z10	l_Z23	1l_Z71	Trait
*R1B-14*	0.000	0.006	0.000	0.824	2.172	5.229	3.564	6.575	9.202	DMPE_GMP
*Natt-4-1*	1.493	1.036	1.158	2.832	1.489	0.000	7.024	0.517	0.161	DMPE_GMP
*Natt-4*	3.396	1.489	2.015	4.067	1.312	0.180	10.901	1.436	0.085	DMPE_GMP
*OHP1*	0.165	0.388	0.183	7.213	9.415	30.780	34.234	54.487	59.068	DMPE_GMP
*PLP3*	1.862	3.792	3.220	6.534	11.013	0.186	4.436	2.671	0.394	DMPE_GMP
*Unch1*	0.672	3.242	1.938	0.889	0.691	0.444	0.306	0.389	0.000	DMPE_GMP
*LEA6*	1.591	1.728	1.103	0.250	7.076	0.000	0.000	0.000	4.464	DMPE_NN
*Unch2*	0.563	0.679	0.689	1.315	0.750	0.699	0.407	0.368	0.736	DRBAE_GMP
*PP2C43*	0.168	0.117	0.053	0.089	0.257	0.500	1.341	0.166	0.022	DRBAE_NN
*RH35*	7.125	7.116	7.990	13.027	9.720	9.384	6.435	7.264	7.859	DRBAE_NN
*GTE9*	5.322	7.109	7.846	6.216	5.283	4.435	4.714	8.335	19.057	DRBAE_NN
*At1g51810*	4.510	4.555	4.111	0.121	0.048	0.232	0.102	0.112	0.019	DRBAE_NN
*APP2*	1.454	1.593	2.142	2.812	2.606	5.192	7.806	7.530	7.688	DRBAE_TOL
*DLGT*	0.628	0.286	0.348	0.540	0.117	0.000	0.033	0.015	2.769	DRBAE_TOL
*Unch3*	4.486	3.225	3.669	4.618	4.227	5.394	4.427	2.965	4.739	DRBAE_TOL
*MAP3K3*	9.770	6.442	6.832	7.638	15.498	7.439	3.624	8.966	13.054	DRBM_NN
*MRS2-F*	3.796	4.135	4.708	4.368	5.286	5.423	2.439	2.440	5.939	DRBM_NN
*ATL1*	0.921	1.066	0.673	0.660	1.487	0.639	0.414	0.384	0.167	DRBM_NN
*CYPRO4*	7.915	6.485	6.757	13.799	3.604	0.241	4.331	3.651	0.101	DRBM_NN
*Unch4*	0.424	0.323	0.505	4.810	4.168	10.370	28.037	6.092	6.392	DRBM_NN
*Unch5*	7.382	6.814	7.773	7.379	7.778	7.338	3.637	3.644	3.275	DRBM_NN
*H3.2*	80.173	73.105	79.912	135.335	40.979	0.137	61.078	42.911	0.094	DRBM_TOL
*H2B.2*	68.203	57.551	58.994	81.959	27.696	0.503	33.577	24.505	0.191	DRBM_TOL
*Rf1*	0.647	0.982	0.779	1.079	0.498	0.102	0.148	0.229	0.169	DRBM_TOL
*CRPK1*	9.682	8.052	9.137	1.729	7.699	1.734	1.155	3.763	5.077	DRBM_TOL
*Unch6*	115.476	95.787	90.718	92.614	56.059	59.795	37.201	15.995	2.130	DRBM_TOL
*Unch7*	2.452	1.998	3.806	1.117	0.796	1.387	0.176	0.866	0.587	DRBM_TOL
*TsaC*	3.429	2.657	2.564	5.422	1.390	0.821	2.663	2.707	0.532	DRSWR_GMP
*Unch8*	0.000	0.000	0.000	0.670	3.039	0.000	0.000	0.000	0.000	DRSWR_GMP
*Unch9*	2.057	1.522	1.683	4.950	3.236	3.894	9.131	5.389	2.384	DRSWR_GMP
*THT2*	3.391	5.864	3.836	0.086	1.926	0.069	0.026	0.012	0.423	DRSWR_NN
*PHB3*	62.342	53.594	58.896	57.803	22.985	7.935	34.035	15.560	6.054	DRSWR_NN
*LRK*	0.797	1.413	1.430	1.692	1.355	0.144	1.186	1.481	1.662	DRSWR_NN
*Zlp*	3.615	9.415	7.801	0.280	1.022	0.000	2.630	1.542	0.019	DRSWR_NN
*RPS27*	48.187	27.998	48.763	60.575	25.814	20.614	51.447	35.922	9.213	DRSWR_NN
*Unch10*	3.901	7.126	6.150	0.482	0.797	0.000	0.000	0.000	0.000	DRSWR_NN
*SUD1*	19.976	18.915	17.933	9.248	3.823	9.965	9.658	2.316	0.023	DRW_TOL
*MT4A*	183.847	245.697	312.993	0.000	0.000	0.000	0.000	0.396	0.366	DRW_TOL
*BHLH112*	2.758	11.491	11.606	2.419	7.329	1.293	0.329	0.839	1.281	DRW_TOL
*F3H-3*	2.483	0.500	0.418	0.190	0.038	0.249	0.140	0.295	0.142	DRW_TOL
*PILS7*	2.810	0.340	0.531	0.105	0.803	0.034	0.262	1.121	5.839	DRW_TOL
*ZFP8*	0.042	0.209	0.239	1.350	1.631	0.173	6.975	7.526	0.367	DRW_TOL
*RUP2*	0.357	0.099	0.129	0.239	0.190	0.151	4.191	1.914	0.504	DRW_TOL
*HSP26.2*	2.621	2.660	2.459	0.389	0.587	0.627	0.000	0.431	0.421	DRW_TOL
*Unch11*	11.657	9.433	10.577	20.795	31.971	37.048	72.785	31.277	34.098	DRW_TOL
*Unch12*	1.550	0.738	0.866	5.837	3.323	3.748	9.470	4.519	1.269	DRW_TOL
*Q salvage*	4.122	2.144	2.098	3.904	0.553	0.930	6.428	3.281	0.567	DSBAE_NN
*CPP1*	0.802	1.059	1.176	1.706	1.344	1.272	0.747	0.584	0.393	DSBAE_NN
*SnRK1β3*	12.713	10.543	10.737	13.181	12.438	10.015	5.850	5.636	5.533	FRBAE_LN
*TM9SF3*	14.133	15.647	16.469	13.641	16.360	10.906	5.150	6.413	7.402	FRBAE_LN
*Unch13*	0.009	0.005	0.018	0.019	0.000	0.000	0.035	0.245	3.241	FRBAE_LN
*TAF6*	8.913	8.337	9.421	15.841	8.678	4.681	4.012	5.504	4.998	FRSWR_LN
*TaACR11*	18.098	12.946	13.932	14.715	38.872	129.467	127.090	145.918	200.103	FRSWR_LN
*INRPK1*	0.000	0.086	0.106	0.000	0.000	0.051	0.000	0.144	2.713	FRSWR_TOL
*TaAPY6*	3.029	4.155	4.656	5.676	4.720	4.139	1.922	3.351	4.303	FRSWR_TOL
*Unch14*	0.101	0.296	0.241	0.655	1.340	0.000	0.191	0.037	0.019	FRSWR_TOL
*Unch15*	0.796	1.882	2.084	6.287	8.348	1.285	2.815	2.013	3.846	FRSWR_TOL
*EMC1*	32.256	24.992	26.440	40.676	28.450	17.311	14.782	12.352	10.956	FRW_TOL
*JMJ18*	2.839	2.496	2.677	4.028	2.912	1.479	1.570	1.674	1.840	FRW_TOL
*SURF2L*	4.738	4.317	4.759	7.121	2.995	2.346	2.015	3.058	1.750	FRW_TOL
*IAA14*	1.026	1.952	1.983	9.525	7.309	5.014	4.845	7.976	9.073	FRW_TOL
*EIX2*	0.027	0.034	0.069	0.216	0.307	0.264	0.064	0.741	3.466	FRW_TOL
*iPGM*	0.052	0.263	0.211	0.007	1.157	0.139	0.039	0.013	0.484	FSW_Mean
*GAUT7*	2.023	1.596	1.510	3.176	1.476	0.826	1.307	0.822	0.112	FSW_Mean
*SFR2*	0.094	0.066	0.041	2.658	5.094	10.467	11.234	21.043	21.545	FSW_Mean
*SCE1*	80.658	96.090	88.164	58.266	93.706	56.060	26.153	37.886	40.572	FSW_Mean
*MADS50*	0.637	3.987	4.121	3.206	3.920	4.616	0.064	2.559	8.877	FSW_Mean
*TCP5*	0.000	0.000	0.000	2.402	0.719	0.139	2.715	2.717	0.271	GI_LN
*ACA7*	1.154	0.832	0.914	0.017	1.316	0.016	0.080	0.133	0.088	GI_LN
*AIS1*	0.076	0.123	0.080	3.394	3.216	5.204	3.114	2.061	4.954	GI_LN
*PUB42*	3.232	1.986	1.955	1.577	4.058	1.608	0.947	1.802	3.730	GI_LN
*AVT6A*	10.632	11.330	12.367	8.716	16.632	24.682	3.761	7.853	25.453	GI_LN
*PI4KG7*	2.864	2.045	2.182	5.250	5.618	3.722	2.141	2.408	0.338	GI_LN
*Unch16*	8.438	5.156	5.203	3.194	3.824	3.666	5.245	2.322	1.285	GI_LN
*Unch17*	0.000	0.102	0.139	0.007	0.041	0.024	0.045	1.722	1.804	GI_LN
*MgPMT*	0.021	0.174	0.153	3.056	4.779	17.389	33.654	15.805	13.466	RL_NN
*RALF33*	1.200	3.137	4.228	0.108	0.207	0.000	0.320	0.131	0.000	RL_TOL
*HIPP36*	2.445	6.118	6.024	1.362	9.639	27.927	0.530	1.936	2.869	RL_TOL
*Unch18*	2.269	2.321	2.350	2.310	1.928	1.345	1.292	1.242	0.257	RL_TOL
*NIPA4*	4.799	6.599	5.990	8.666	6.755	3.571	2.876	3.589	3.958	RLSR_NN
*PHR1*	1.841	1.256	1.503	3.863	2.168	0.008	0.265	0.663	0.005	RLSR_NN
*MGAT1*	3.710	3.445	3.836	4.955	4.345	3.320	1.804	2.840	4.869	RLSR_NN
*GSTU*	0.048	1.210	1.319	0.098	0.147	0.036	0.137	0.135	0.115	RLSR_NN
*At5g03900*	4.378	4.120	5.052	9.260	7.996	11.176	16.966	18.133	24.673	RLSR_NN
*PUM1*	5.366	3.337	4.259	7.815	2.594	0.441	1.570	1.756	0.115	RPC_Mean
*RGA5*	0.726	1.311	1.346	1.174	1.226	1.106	0.415	0.971	1.602	RSLR_GMP
*FRO7*	0.066	0.334	0.340	0.491	1.161	13.412	0.268	8.731	73.538	RSLR_GMP
*β-1,3-GalT12*	4.291	4.180	3.957	7.031	5.770	3.354	3.002	2.208	1.261	RSLR_LN
*NHL13*	72.143	49.747	50.802	24.744	72.413	35.535	18.135	6.775	7.139	RSLR_LN
*SDA1*	4.795	4.066	4.851	8.357	3.118	1.673	2.215	3.055	1.502	RSLR_LN
*BGLU16*	52.647	42.467	45.717	2.595	12.242	44.378	3.307	2.066	58.601	RSLR_LN
*Unch19*	8.522	18.979	15.754	5.973	20.692	5.400	2.656	8.910	11.398	RSLR_LN
*bHLH62*	3.967	8.124	7.725	1.925	2.420	2.673	0.740	0.597	0.008	RSLR_TOL
*DRM1*	137.297	100.093	100.491	26.069	259.020	195.518	32.614	13.572	53.597	RSLR_TOL
*D27*	0.000	0.000	0.000	0.764	0.970	7.326	8.316	25.662	55.139	RSLR_TOL
*nod-93*	15.040	3.594	3.394	2.878	4.763	0.415	5.417	0.175	0.000	SL_Mean
*nod-93-1*	11.781	2.382	2.611	1.070	3.935	0.174	2.866	0.000	0.037	SL_Mean
*TaNAR1*	6.346	9.824	6.919	19.188	9.958	9.958	15.459	18.140	16.062	SL_Mean
*PHR3*	0.000	0.255	0.109	0.056	0.039	0.096	0.000	0.565	0.000	SL_Mean
*AIP1-1*	18.140	16.062	15.459	19.188	9.958	9.958	9.824	6.919	6.346	SL_NN
*GSTU6*	0.057	0.358	0.452	0.065	0.352	0.255	0.000	0.041	0.135	SL_NN
*SALT*	6.190	6.408	7.991	0.039	0.000	0.000	0.022	0.000	0.012	SWC_Mean
*TIM8*	79.953	63.650	61.885	77.300	22.361	7.092	19.864	23.946	6.156	SWC_Mean
*At5g02860*	0.000	0.475	0.191	1.359	0.999	4.465	6.299	5.082	1.954	SWC_NN
*At4g02110*	1.970	1.360	1.225	3.768	1.071	0.889	0.679	0.718	0.371	SWC_NN
*MADS5*	0.281	1.452	1.444	0.265	0.925	16.238	1.323	0.435	11.765	SWC_NN
*Unch20*	2.011	2.567	2.108	5.249	2.627	0.722	1.244	1.093	0.013	SWC_NN
*SRL2*	10.499	9.060	8.739	30.206	13.107	5.197	7.323	7.382	2.579	TBM_Mean
*DDX24*	2.730	3.581	3.336	4.273	3.139	2.507	1.991	2.510	2.582	TBM_Mean
*UBX4*	0.743	1.650	0.997	0.570	0.454	3.289	0.596	1.638	3.007	TBM_Mean
*F-box*	0.411	0.599	0.509	2.510	2.356	2.478	1.329	1.488	1.232	TBM_Mean
*PSMA3*	61.523	62.969	56.565	73.509	54.961	28.967	34.083	24.820	23.574	TBM_Mean
*SBE1*	0.590	0.440	0.448	3.982	1.611	0.499	8.774	2.674	0.707	TBM_Mean
*Unch21*	1.416	3.683	6.367	3.085	5.853	9.971	0.641	5.642	18.468	TBM_Mean
*Unch22*	2.999	3.071	3.158	1.963	2.754	0.762	0.278	0.184	0.040	TBM_Mean
*FH5*	5.061	5.414	6.614	9.007	7.787	3.091	3.012	3.356	1.514	TDM_NN
*ADT2*	0.982	0.613	0.460	2.888	1.879	4.091	5.233	1.958	1.597	TDM_NN
*RbcX2*	0.137	0.095	0.208	0.531	1.097	19.929	5.301	71.257	250.186	TDM_NN
*OTU9*	6.771	3.037	3.457	2.439	4.374	2.352	1.251	2.133	4.495	TDM_NN
*Unch23*	5.772	4.874	5.955	5.767	6.864	7.033	2.747	2.752	5.586	TDM_NN
*Unch24*	0.493	0.393	0.529	2.811	4.057	23.070	32.137	17.030	23.061	TDM_NN
*At5g63440*	5.099	5.572	5.877	10.175	6.095	5.105	4.295	3.866	2.734	TMC_Mean
*AVT6D*	18.452	7.147	7.986	8.885	16.810	0.661	11.961	3.455	3.022	TMC_Mean
*GDSL-lipase*	0.408	0.751	0.714	0.820	1.658	2.186	0.690	0.705	0.402	TMC_Mean
*SNF1β1*	22.562	20.825	22.516	20.186	20.674	15.025	6.677	10.153	22.988	TMC_Mean
*TGT*	1.141	1.282	1.366	1.429	1.000	1.595	0.652	0.961	0.488	TMC_Mean
*KLR1*	27.084	28.845	31.156	25.888	21.240	32.478	10.779	9.502	8.803	TMC_Mean
*FPF1-4*	0.000	0.000	0.000	0.000	0.000	0.000	0.044	7.544	0.444	TMC_Mean
*TSM1*	208.601	257.902	247.046	88.232	169.307	460.583	42.320	19.890	7.349	TMC_Mean
*TSM1-1*	57.995	36.580	38.919	2.715	2.460	0.295	1.558	0.415	0.419	TMC_Mean

Whereas r, root; s, stem; l, leaf.

### Allelic effects of significant SNPs on respective phenotypes

**Figure 9:**
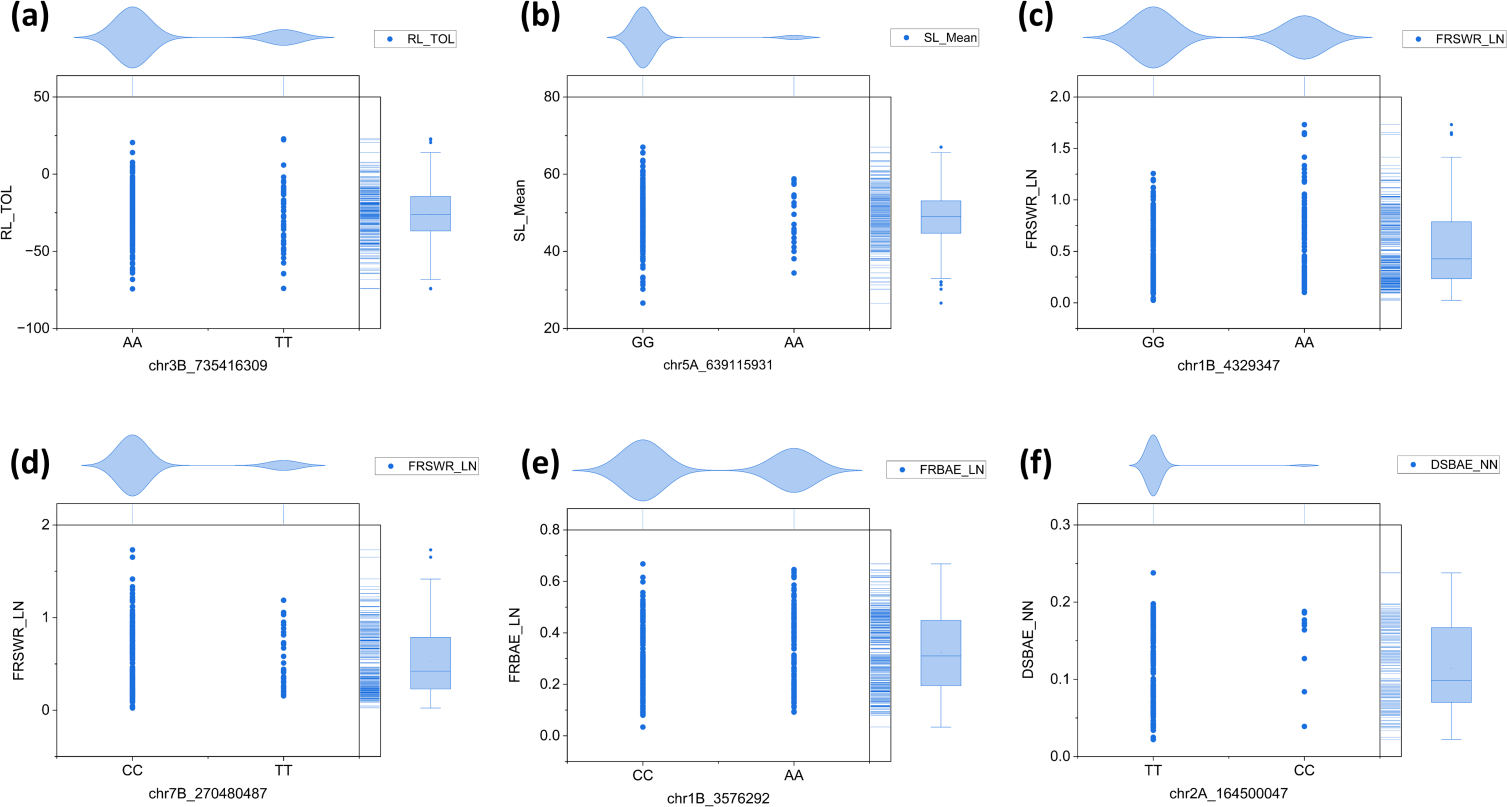
Haplotype analysis of six SNPs associated with RL, SL_, FRSWR, DSBAE, and FRBAE traits. **(a)** chr3B_735416309, with the AA allele dominating in RL_TOL. **(b)** chr5A_639115931, with the GG allele dominating in SL_Mean. **(c)** chr1B_4329347, with the GG allele dominant in the associated traits. **(d)** chr7B_270480487, with the CC allele dominating in FRSWR_LN. **(e)** chr1B_3576292, with the CC allele dominating in FRBAE_LN. **(f)** chr2A_164500047, with the TT allele dominant in DSBAE_NN.

We conducted haplotype analysis on the six key SNPs associated with the RL_TOL, SL_Mean, FRSWR_LN, DSBAE, and FRBAE traits. For chr3B_735416309, the alleles were AA and TT, with the AA allele being dominant in RL_TOL (**Figure 9a**). Similarly, for chr5A_639115931, the alleles were GG and AA, with the GG allele dominating in SL_Mean (**Figure 9b**). For chr1B_4329347, the alleles were GG and AA, with GG being the dominant allele, while for chr7B_270480487, the alleles were CC and TT, with CC dominating in FRSWR_LN (**Figures 9c, d**). Additionally, for chr1B_3576292, the alleles were CC and AA, with CC dominating in FRBAE_LN (**Figure 9e**). Finally, for chr2A_164500047, the alleles were TT and CC, with TT being dominant in DSBAE_NN (**Figure 9f**). The haplotype analysis was performed using the data presented in **Supplementary Table S7**.

## Discussion

**Figure 10:**
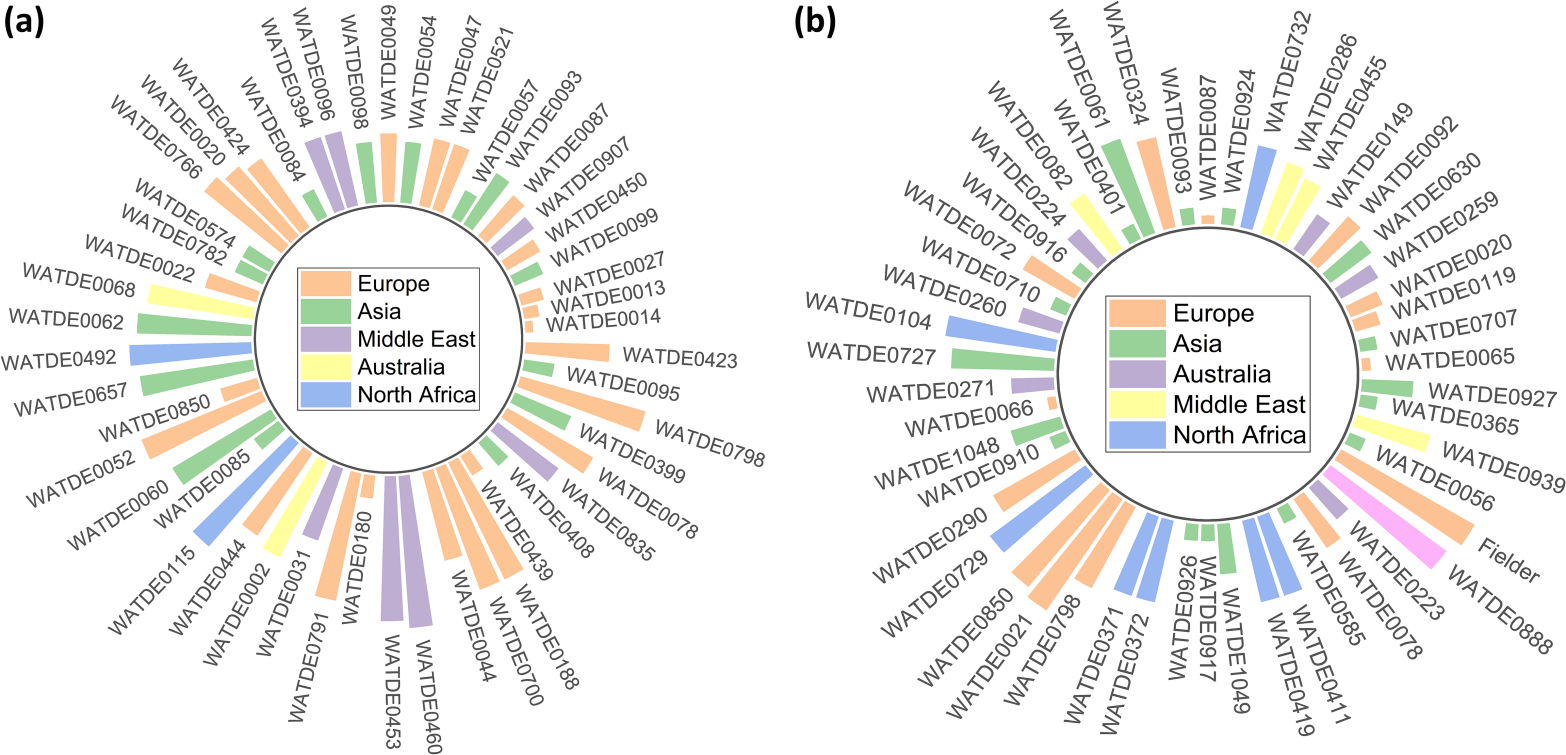
Differential performance of European wheat landraces and Eurasian landraces under contrasting nitrogen regimes. **(a)** European landraces demonstrated superior growth under low nitrogen (LN) conditions, while **(b)** Eurasian accessions exhibited enhanced performance under normal nitrogen (NN) availability, reflecting adaptive genetic diversity shaped by regional agroecological selection pressures.

Early development of plants is critical for providing a strong base for further growth. The seedling stage root-shoot biomass has been extensively studied in wheat, with its potential relation to nitrogen utilization and productivity (Gavito et al., 2001; Foulkes et al., 2009; Cormier et al., 2016; Huang et al., 2024). Several studies (Gaju et al., 2011; Allard et al., 2013; Lu et al., 2015; Zhang et al., 2015; Hawkesford, 2017; Sprunger et al., 2018; De Oliveira Silva et al., 2020) have been conducted on root-shoot biomass dynamics under varying nitrogen conditions, but Watkins wheat landraces have not been widely studied in this context. Wheat selection and breeding programs have traditionally focused on aboveground traits and yield, often overlooking the impact on root traits (Narayanan and Vara Prasad, 2014). Investigating the root-shoot biomass related traits is also of significance to major characteristics, viz., SL, RL, FSW, FRW, FRSWR, FSBAE, & DSBAE (Nakhforoosh et al., 2014; Narayanan et al., 2014; Bektas et al., 2016; Xie et al., 2017; Junaidi et al., 2018). The bread wheat landraces referenced in this study were initially documented by Arthur Ernest Watkins in his seminal work, The Wheat Species: A Critique, published in 1930 (Watkins, 1930; Wingen et al., 2014). The identification of key genes influencing root-shoot-ground biomass traits in Watkins wheat landraces has provided valuable insights into their genetic regulation under two nitrogen treatments. In particular, several significant genes have been identified through GWAS, with their roles being further confirmed by gene expression seedling traits (Beyer et al., 2019; Yang et al., 2020; Jung et al., 2021; Javid et al., 2022). The screening of landraces based on the highest total biomass under both LN and NN conditions revealed significant correlations with various growth-related traits, providing insights into the factors driving biomass accumulation. TBM exhibits strong positive relationships with SL (0.458***), FSW (0.895***), DSW (0.711***), and TDM (0.759***), indicating that shoot-related traits are primary contributors to biomass production. However, TBM shows significant negative correlations with RL (-0.145***) and RSLR (-0.257***), suggesting a trade-off between shoot growth and root elongation. Interestingly, the correlation patterns for traits like FRSWR and DRSWR vary between LN and NN conditions, with LN showing positive trends and NN showing negative trends, highlighting the influence of nitrogen availability on biomass allocation. TBM also positively correlates with DSBAE (0.107**) and GI (0.149***), emphasizing the role of dry shoot in enhancing biomass. Weak negative correlations with traits such as WHCI (-0.069) and RPC (-0.113**) suggest that higher biomass may come at the cost of reduced water retention and root efficiency. These findings underscore the importance of balancing root-shoot traits to optimize biomass production under varying environmental conditions. The PCA and hierarchical cluster analysis (HCA) revealed distinct patterns of trait relationships and grouping among landraces, offering a comprehensive understanding of the underlying genetic structure and adaptability of landraces to varying nitrogen availability (Padhi et al., 2023). The PCA revealed significant correlations among traits, with the first four principal components explaining 86.642% of the total variance. Principal Component 1 (Prin1), which accounted for 41.144% of the variance, highlighted a strong contrast between traits such as DRBM, DRSWR, and SMF (positively correlated) and DSBAE, DSW, and DMPE (negatively correlated). This contrast likely reflects trade-offs in resource allocation under nitrogen stress, where certain traits associated with biomass production and stress tolerance are prioritized over others (Maire et al., 2009; Lynch, 2019). For instance, the positive correlation of DRBM and DRSWR with Prin1 suggests that these traits may be key indicators of nitrogen use efficiency or stress resilience. Conversely, the negative correlation of DSBAE and DSW with Prin1 may indicate reduced investment in these traits under nitrogen-limited conditions. These findings are consistent with previous research that has identified similar trade-offs in crop responses to nutrient stress (Maire et al., 2009; Lynch, 2019; He et al., 2022). PC1 PC2, PC3, and PC4 further elucidated additional dimensions of trait variability (De Souza et al., 2023). Prin2 highlighted positive correlations among traits such as DRW, TBM, and TMC, which may reflect growth-related processes under NN conditions. In contrast, Prin3 and Prin4 captured contrasting relationships among traits like DSBAE, DMPE, and WHCI, suggesting distinct physiological mechanisms underlying nitrogen adaptation. Together, these principal components provide a robust framework for understanding the complex interplay of traits influencing nitrogen efficiency and stress tolerance (Ding et al., 2023). The HCA supported the PCA findings by revealing four distinct clusters of landraces under both LN and NN conditions (Vaezi et al., 2019). The use of Ward’s method and Euclidean distance effectively captured the genetic diversity among landraces, as evidenced by the clear separation of clusters in the dendrogram and constellation plots (Ward, 1963; Abdel-Sattar et al., 2024). Garcia et al., 2021 (Garcia et al., 2021) suggested that the presence of subgroups within the main clusters in yellow, blue, green, and red subgroups suggests that landraces within these groups may share similar genetic or physiological mechanisms for nitrogen adaptation. In our study, landraces under LN conditions demonstrate diverse adaptive strategies, with some exhibiting superior biomass production and stress tolerance, while others reflect alternative mechanisms of adaptation likely influenced by agroecological variations such as soil nitrogen availability, moisture levels, and local climatic conditions. Based on their consistent presence across all traits, the following landraces are recommended for their superior performance under LN conditions: WATDE0014, WATDE0013, WATDE0027, WATDE0907, WATDE0087, WATDE0057, WATDE0521, WATDE0093, WATDE0450, WATDE0099, WATDE0180, WATDE0047, WATDE0424, WATDE0020, and WATDE0052 (**Figure 10A**). For NN conditions, the recommended landraces are: WATDE0065, WATDE0707, WATDE0119, WATDE0020, WATDE0259, WATDE0630, WATDE0092, WATDE0149, WATDE0455, WATDE0286, WATDE0732, WATDE0087, WATDE0324, WATDE0093, and WATDE0061 (**Figure 10B**). These landraces, predominantly from Europe and Asia, demonstrate adaptability to varying agroecological zones, characterized by differences in nitrogen availability and climatic stressors. Their strong performance in both root and shoot growth under NN conditions, combined with their ability to sustain growth in nitrogen-limited environments, highlights their potential as resilient candidates for breeding programs. By optimizing root-shoot allocation, these landraces can contribute to sustainable agriculture, particularly in regions with limited nitrogen availability, by improving productivity while reducing dependency on synthetic fertilizers. The current study identified several key genes with significant roles in regulating root-shoot biomass development in wheat, highlighting their potential contributions to leaf, stem, and root growth. For leaf growth, the gene *OHP1* exhibited the highest expression levels across different developmental stages (Z10, Z23, Z71), with values of 34.234, 54.487, and 59.068, respectively. This strong expression suggests that *OHP1*, a light-harvesting complex-like protein, plays a critical role in photosynthesis and protein binding, which are essential for above-ground biomass production. This study also revealed that *OHP1*, a light-harvesting complex-like protein located in the chloroplast, plays a crucial role in protein binding, which is essential for maintaining the efficiency of photosynthesis and directly influences above-ground biomass. Curtis et al., 2019 (Curtis et al., 2019) also reported that the OHP1/BLZ1/bZIP63 transcription factor gene was expressed at similar levels in both endosperm and embryo, and clearly its expression in the embryo suggests that regulating storage protein gene expression is not its only role. In the context of stem growth, the gene *PHR3* showed notable expression in root tissues, with values of 6.346, 9.824, and 6.919 in r_Z10, r_Z13, and r_Z39, respectively. Although the expression data is from root tissues, *PHR3*’s role in phosphate homeostasis and nutrient signaling is likely crucial for stem development, as phosphate is vital for energy transfer and structural integrity in stems. For root growth, multiple genes demonstrated dominant expression, underscoring their importance in below-ground biomass development. Zheng et al., 2020 (Zheng et al., 2020) already reported that transgenic wheat lines with down-regulation of *TaPHR3-A1* exhibited retarded growth and root hair development at the seedling stage and showed yield-related effects at the adult stage when grown in both low- and sufficient Pi conditions, indicating that *TaPHR3-A1* positively regulated tolerance to low Pi. Ren et al., 2025 (Ren et al., 2025) reported that in UV-treated rice, *PHR1* and *PHR2* were significantly reduced, while *PHR3* and *RLI1* stayed the same. *PHR4* was also lowered under both +P and –P conditions. We identified that the quinone salvage enzyme (*Q salvage*) is involved in quinone metabolism and redox homeostasis, critical for managing oxidative stress and maintaining cellular redox balance in both root-shoot-ground biomass. The gene *Q salvage* showed high expression in roots, with values of 4.122, 2.144, and 2.098 in r_Z10, r_Z13, and r_Z39, respectively, indicating its role in quinone metabolism and redox homeostasis, which are critical for root health and stress tolerance (Xu et al., 2015). Similarly, *SnRK1β3* exhibited the highest expression in roots for the trait FRBAE_LN, with values of 14.133, 15.647, and 16.469, highlighting its involvement in energy sensing and stress responses (Margalha et al., 2019). Our findings indicate that *SnRK1β3*, involved in energy sensing and stress responses, regulates energy allocation between root-shoot biomass, especially under stress conditions (Van Leene et al., 2022; Persyn et al., 2024).

Due to likely redundancy, future loss-of-function studies of *SnRK1β* will require the generation of double (and triple) knockouts or, in the case of lethality, transient or conditional/inducible knock-down (Ramon et al., 2019). We found that *TAF6*, crucial for transcriptional regulation and RNA polymerase II assembly, influences the expression of genes involved in biomass production, affecting both genotypic and phenotypic traits (Li et al., 2023). Parvathi et al., 2017 (Parvathi and Nataraja, 2017) stated that RTPCR analysis in leaf tissues of finger millet experiencing different levels of drought revealed its stress responsiveness in three different landraces. The gene is also induced under salt, osmotic, and oxidative stresses at the seedling stage. *TaACR11*, with expression values of 18.098, 12.946, and 13.932, plays a key role in detoxification and stress tolerance, essential for maintaining root health. This study revealed that *TaACR11*, involved in detoxification and stress tolerance, is essential for maintaining below-ground biomass health by protecting roots from toxic compounds and environmental stresses (Ma, 2010). Additionally, *INRPK1* showed remarkable expression in roots for the trait FRSWR_TOL, with values of 80.658, 96.090, and 88.164, emphasizing its role in inositol phosphate metabolism and signaling, which are vital for root development and stress adaptation. We identified that *INRPK1*, playing a role in inositol phosphate metabolism and signaling, is important for cellular pathways regulating biomass development and stress responses (Thomson et al., 2006; Koppolu et al., 2013). Bassett et al., 2000 (Bassett et al., 2000) reported in their findings that the expression of *INRPK1* in roots would be regulated at three levels: transcription initiation, mRNA processing, and translation initiation. Further our findings indicate that *TaAPY6*, involved in nucleotide metabolism and stress responses, is crucial for managing energy resources and stress signaling vital for biomass growth. *TaAPY6*, associated with purinergic signaling, is linked to stress-responsive growth adjustments (Chowdhury et al., 2023). Liu et al., 2019 (Liu et al., 2019) applied mannitol treatment to produce an artificial drought stress condition in the wheat seedlings. Their results showed that all the TaAPYs could be up-regulated within 24 h, among which *TaAPY1*, *TaAPY3-4*, and *TaAPY6* reached an extremely high expression level in the leaves. Zhang et al., 2023 (Zhang et al., 2023) reported that *RALF36* is relatively more potent than *RALF33* in inhibiting root length in wheat. We found that *RALF33* is involved in cell wall signaling and growth regulation, plays a significant role in the expansion and strengthening of cell walls, and is essential for the structural integrity of biomass (Xue et al., 2024). This study revealed that *TaNAR1*, crucial for nitrate assimilation and nutrient metabolism, enhances the plant’s ability to uptake and utilize nitrogen, a key nutrient for below-ground biomass (Zhao et al., 2013). Rather than relying solely on percentage changes, we now emphasize the relative ranking of effect sizes across all tested SNPs. Both *RALF33* and *TaNAR1* were in the top tier of effect magnitudes for root and shoot growth, distinguishing them from variants with weaker or less consistent effects. Our findings indicate that *PHB3*, involved in mitochondrial function, stress responses, and cell proliferation, is crucial for maintaining cellular energy production and stress resilience. Li et al., 2022 (Li et al., 2022) reported that the expression of *PHB3* in roots using histochemical β-glucuronidase analysis and found that *PHB3* was strongly expressed in the different stages of lateral root primordia. In Duan et al., 2021 (Duan et al., 2021) results showed that *FH5* was coupled to actin depolymerizing factor 9, implying that *FH5* may play a key role in the cell growth and development of rice via depolymerization of the cytoskeleton. We found that *FH5*, involved in cytoskeleton organization and cell growth, plays a significant role in cell division and expansion, essential for biomass development. This study revealed that *MGAT1*, involved in glycosylation and protein processing, is important for the proper folding and function of proteins critical for biomass growth. Abdullah et al., 2016 (Abdullah et al., 2016) stated in their results that the *MGAT1* gene showed maximum expression at an earlier stage, thus supporting the fact that these two genes may play roles in lipid metabolism during the period prior to oil deposition in seeds. Collectively, these genes play a significant role in regulating the genotypic and phenotypic traits related to root-shoot biomass competition, ensuring optimal plant growth and stress resilience (Zaffar et al., 2024). While this study provides valuable insights, but future research could integrate omics approaches to elucidate the molecular mechanisms underlying the observed trait correlations and landrace groupings, offering a more comprehensive understanding of nitrogen use efficiency and stress resilience in wheat.

## Conclusion

This study integrates phenotypic and genotypic analyses to unravel the competitive dynamics between root and shoot biomass in Watkins wheat landraces under low-nitrogen (LN) and normal-nitrogen (NN) conditions. Our findings underscore trade-offs between root and shoot traits, with strong correlations between total biomass and shoot-related traits, and negative correlations with root-related traits under LN conditions. Regional ranking through PCA and cluster analysis identified elite landraces which demonstrate superior adaptability and biomass production under LN conditions. These landraces represent valuable genetic resources that can be integrated into breeding pipelines to enhance nitrogen use efficiency and stress resilience in wheat. Additionally, GWAS revealed key SNPs and candidate genes, including *RALF33*, linked to biomass traits, providing deeper insights into the genetic basis of nitrogen adaptation. These findings have the potential to accelerate the development of high-performing wheat varieties tailored to diverse nitrogen conditions. However, certain limitations must be acknowledged. The controlled-environment setting of this study may not fully capture the complex interactions observed under field conditions. Future studies integrating multi-omics approaches, such as transcriptomics and metabolomics, alongside field validation across diverse environments, are essential to confirm and expand these findings. Moreover, exploring additional traits, such as root architecture and nutrient uptake efficiency, will provide a more comprehensive understanding of nitrogen adaptation.

## Data Availability

The original contributions presented in the study are included in the article/**Supplementary Material**. Further inquiries can be directed to the corresponding authors.
